# The role of natural outdoor environment on COVID-19 mortality and hospitalisations among older community-dwellers in the pre-vaccination period: the Register RELOC-AGE cohort study in Sweden

**DOI:** 10.1186/s12889-025-25515-w

**Published:** 2025-12-07

**Authors:** Mthabisi Anele Bhebhe, Anton Nilsson, Jonas Björk, Susanne Iwarsson, Giedre Gefenaite

**Affiliations:** 1 Department of Health Sciences, Faculty of Medicine, Lund University, Lund, Sweden; 2 Division of Occupational and Environmental Medicine, Lund University, Lund, Sweden; 3 Department of Translational Medicine, Faculty of Medicine, Lund University, Malmö, Sweden; 4 Clinical Studies Sweden, Forum South, Skåne University Hospital, Lund, Sweden

**Keywords:** Greenness, Living environment, COVID-19 mortality, COVID-19 admissions, Severe COVID-19, Population density

## Abstract

**Background:**

Most COVID-19-related deaths and hospitalisations occurred among older people. Natural outdoor environments influence health outcomes, but the evidence of their effect on COVID-19 outcomes among older adults is limited. This study aimed to investigate associations between residential natural outdoor environment and COVID-19-related deaths or hospitalisations among community dwellers aged 59 years or older, and whether income and education moderated these associations.

**Methods:**

The current study is part of the Register RELCOC-AGE cohort and included ≥ 59-year-old community-dwellers (*N* = 299,219) in Scania, Sweden. The main exposure was the Perceived Sensory Dimension Score (PSD-score), an aggregated area-level score indicating the presence of residential natural outdoor environment, measured between 2008 and 2019, grouped into low, intermediate, and high PSD-score. COVID-19 deaths and hospitalisations were assessed from 1 January to 31 December 2020. Hazard ratios (HRs) were estimated using Cox proportional hazard regression, and moderation by income or education was assessed using interaction terms. Models were adjusted for demographic and socioeconomic characteristics, population density and comorbidities.

**Results:**

The adjusted HRs for COVID-19 deaths and hospitalisations among intermediate– as compared to low PSD-score residents– were 0.90 (95% CI: 0.71–1.15) and 0.88 (95% CI: 0.77–1.01), respectively. Among high PSD-score residents, adjusted HRs were 0.88 (95% CI: 0.62–1.24) and 0.92 (95% CI: 0.76–1.12) for COVID-19 deaths and hospitalisations, respectively. No evidence of moderation of associations by income and education was found.

**Conclusion:**

A protective effect from residential natural outdoor environment on COVID-19 hospitalisation and death was suggested, but statistical uncertainty was substantial and the evidence thus inconclusive. Future investigations across diverse populations could shed more light on the role of residential living environments in mitigating the consequences of epidemics and pandemics.

**Supplementary Information:**

The online version contains supplementary material available at 10.1186/s12889-025-25515-w.

## Background

The Sustainable Development Goal 11, target 11.7, underlines the importance of ensuring inclusive access to outdoor spaces, particularly for older people, women, children and people with disabilities [1]. Evidence, mostly from cross-sectional studies, has suggested that green outdoor environments (defined here as places with ‘natural surfaces’ or ‘natural settings’ or urban greenery [2]) are an important health determinant, affecting health through multiple possible pathways, such as increasing physical activity, providing mental or physical restoration, better air quality, reducing traffic noise and heat-island effects and increasing microbial diversity important for immunoregulation [3–6]. Exposure to green outdoor environments is associated with lower risks of diabetes, obesity, cardiovascular diseases, respiratory diseases and all-cause mortality [2, 7–9].

However, the role of exposure to green outdoor environments on COVID-19 outcomes is, so far, unclear. Heckert and Bristowe [5] conducted a scoping review investigating the possible effects of green outdoor environment exposure on COVID-19 transmission or deaths. There were no systematic findings as to how exposure to green outdoor environments influenced COVID-19 outcomes [5]. Based on aggregated data, a cross-sectional study by Kartal et al., [10] found that mobility to public parks was associated with more COVID-19 deaths. Their study, however, did not report on the potential mechanisms behind this association. Another review [11] found that overall the results were mixed, although several ecological studies in the U. S. as well as the Danish cohort study [12], reported lower COVID-19 mortality in counties with higher residential greenness. Studies on aggregate data are, however, prone to ecological bias [13], which makes it problematic to draw conclusions about individual-level COVID-19 mortality in relation to green outdoor environment exposure.

Moreover, previous research [5, 11] on green outdoor environments and COVID-19 outcomes has focused on the general population. The diverse pandemic experiences of older people suggest a need for research that focuses specifically on this group [14].

Compared to other Nordic countries, Sweden recorded higher incidences of COVID-19-related cases and deaths, particularly among older adults. Between March 2020 and March 2022, individuals aged 70 years or older accounted for 14,703 (88.3%) out of the 16,645 COVID-19 deaths in Sweden, making this group highly overrepresented among the COVID-19 deaths. Moreover, in 2020 alone, out of the 30,448 COVID-19 cases in Sweden, 19,961 (66%) were attributed to adults aged at least 60 years. Some of the factors that contributed to high COVID-19 death rates among older adults were comorbidities (e.g., hypertension, diabetes and lung diseases), age-related decline in functionality or frailty, and residency in nursing homes [15–18]. For instance, among individuals who died from COVID-19 in Sweden, 78% had hypertension, 49% had cardiovascular diseases, 28% had diabetes, 15% had lung disease, and 72% of those aged 70 years or above were residing in nursing homes or receiving in-home care services [19].

There are recognised challenges in establishing the cause-and-effect pathways through which green outdoor environments influence population health. The evidence from cross-sectional studies, pointing out significant associations between green outdoor environment exposure and health, has not been similarly demonstrated using longitudinal studies [4]. Moreover, the methods for assessing green outdoor environment exposure have been inconsistent across studies, contributing to the variation in study findings. Geneshka et al. [4] highlighted that the Normalised Difference Vegetation Index (NDVI) was the most frequently used objective method to measure exposure to green outdoor environments in longitudinal studies.

The main limitation of objective measurement methods is that they might fail to capture important perceptions regarding which qualities of green outdoor environments have health-promoting effects [20–22]. Consequently, there are knowledge gaps concerning which qualities or types of green outdoor environments have an impact on health outcomes [23].

Although in previous studies [20, 24–26], serene, natural, diverse and cohesive were identified as perceived sensory dimensions (PSDs) that describe aesthetic qualities of natural outdoor environment reflecting greenness important for health, the evidence is insufficient as to how PSDs may have influenced COVID-19 outcomes. At the same time, attempts to separately investigate the health effects of each PSD might lead to confounding, as they tend to be associated with each other [20]. Further, it has been suggested [2, 27, 28] that socioeconomic status moderates the association between health and green outdoor environments. For instance, people with low income or education might benefit more from exposure to green outdoor environments than their higher socioeconomic counterparts. While the moderating effects of income and education on the health benefits of exposure to green outdoor environments are not well understood [23], there is emerging evidence that socioeconomic factors play an important role in the association between greenness and COVID-19 outcomes [12]. Focusing on community-dwelling adults aged 59 years or older, this study aimed to investigate the association between residential natural outdoor environment and the risk of COVID-19 death or hospitalisation in the pre-vaccination period, and whether income or education moderated this relationship.

## Methods

### Data sources

This study was part of the RELOC-AGE research project, which aims to study housing, relocations and health in the Swedish population aged 55 years or above [29].

Using the unique personal identity number given to every resident who is born or has resided for at least a year in Sweden [30], eight Swedish population-based registers covering the period from 1987 to 2021 were linked. Specifically, the Swedish Total Population Register (TPR) [31] was linked to the Longitudinal Integrated database for Health Insurance and Labor Market Studies (LISA) [32], the Real Estate Property Register (REPR) and Apartment Registers (AR) [33], the Geographical database [33], the Scania outdoor environment database (ScOut), the National Patient Register (NPR) [34, 35], the Cause of Death Register (DR) [36], and the National Register of Care and Social Services for the Elderly and Persons with Impairments (NRCS). Table A.1 in the appendix provides a detailed presentation of the characteristics of these data sources.

### Study setting and population

**Fig. 1:**
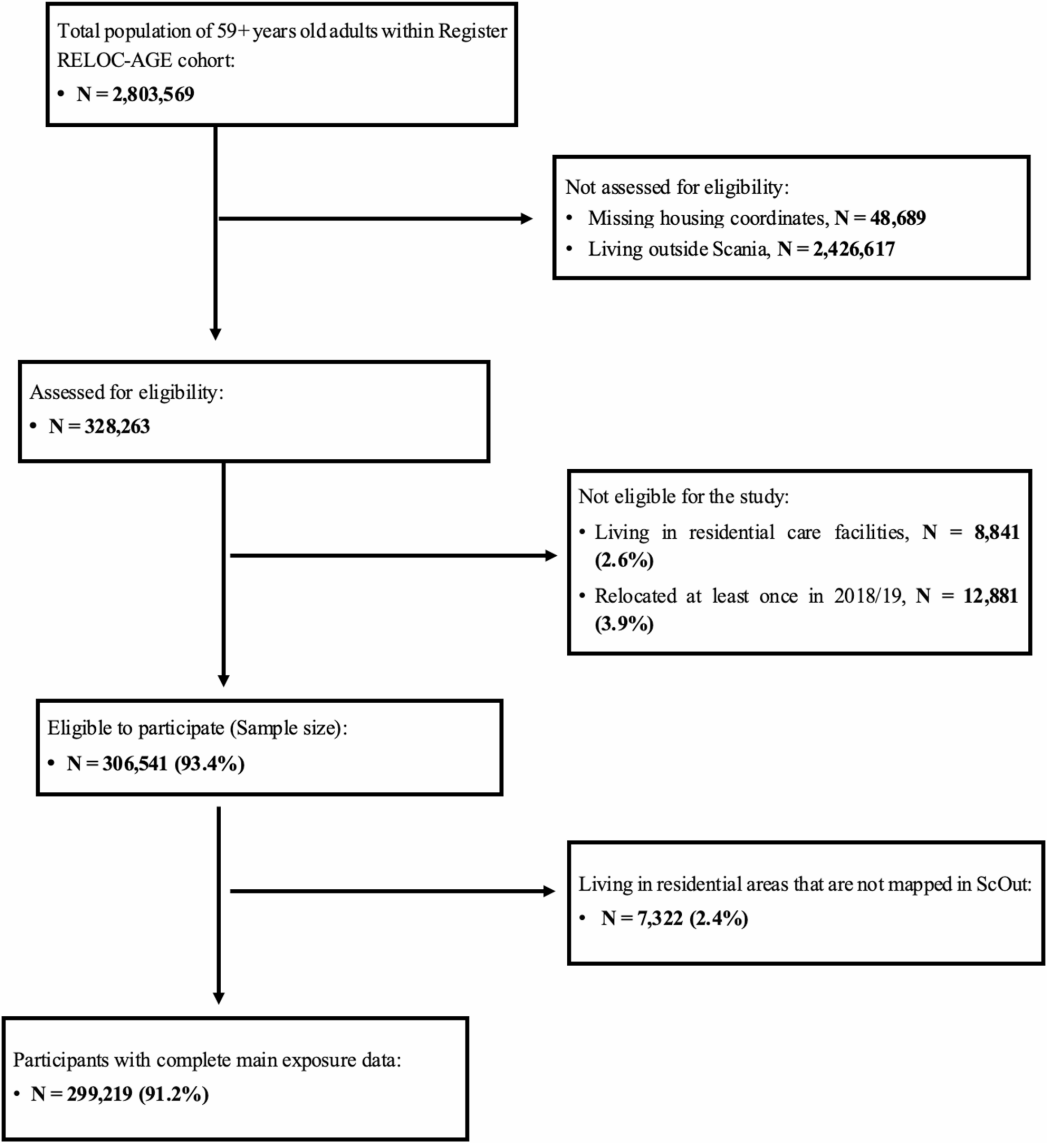
The flowchart of the selection of the study participants

The current study was conducted in Scania, the southernmost administrative county in Sweden, with a population of 1,421,781 people at the end of 2023 [37]. Figure 1 illustrates the selection of the study participants derived from the Register RELOC-AGE cohort, born between 1908 and 1961, and alive on 1 January 2020, as recorded in the TPR (*N* = 2,803,569). Individuals with missing housing coordinates (*N* = 48,689) and those who resided outside Scania in 2020 (*N* = 2,426,617) were excluded and not assessed for eligibility. The Scanian subsample (*N* = 328,263) was then assessed for eligibility, of which those who were in residential care facilities at baseline 2020 were excluded (*N* = 8,841/328,263; 2.6%) because the current study focused on community-dwellers only. Individuals who relocated at least once in 2018 and 2019 (*N* = 12,881/328,263; 3.9%) were excluded as a measure to minimise nondifferential exposure status misclassification bias.

Further, out of those found to be eligible (*N* = 306,541), 7,322 (2.4%) were excluded as living in areas not mapped in the ScOut database. The final study sample included *N* = 299,219 (91.2%) community-dwellers who were followed from 1 January to 31 December 2020.

### Primary outcome

**Fig. 2:**
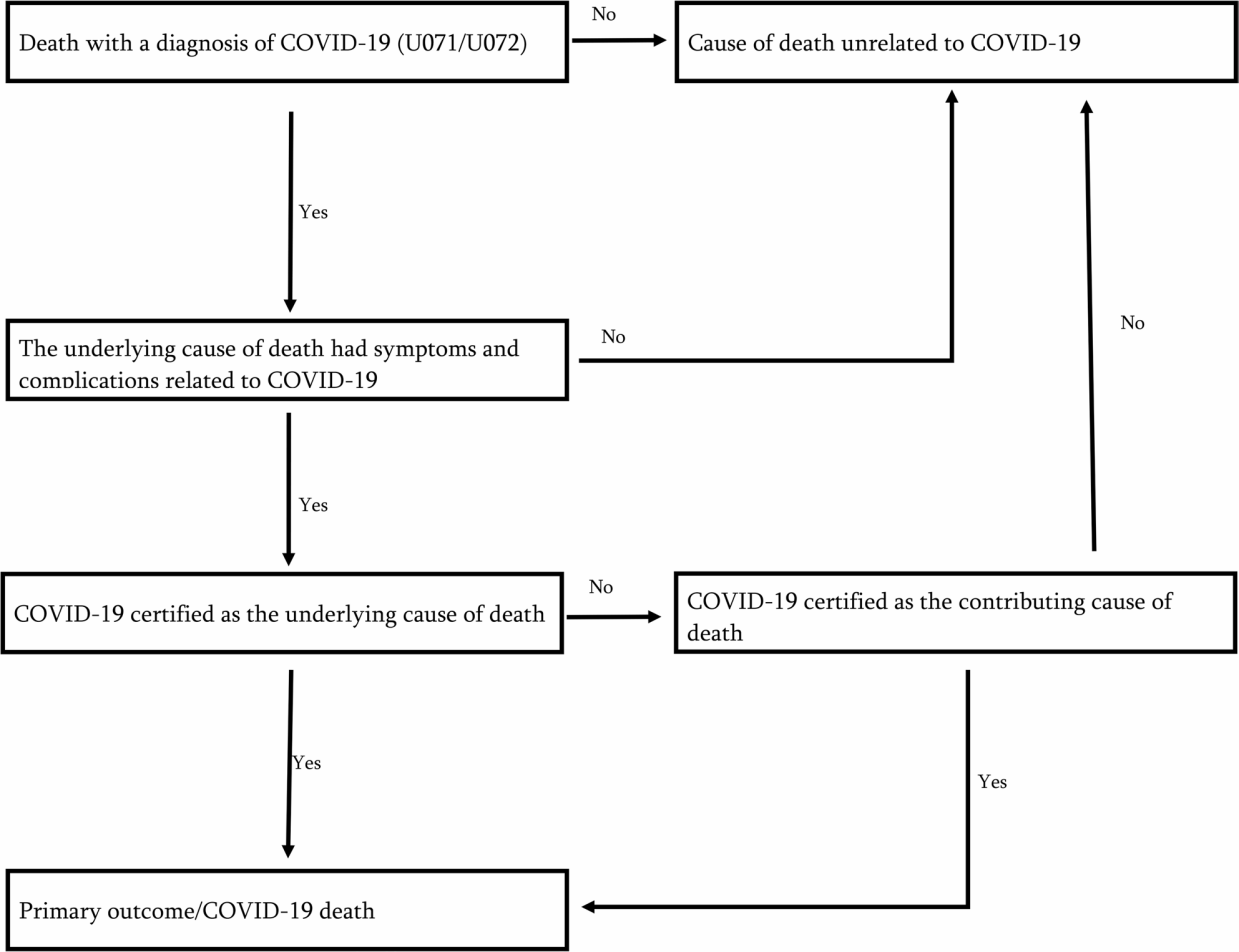
The algorithm used to determine COVID-19 deaths (primary outcome) from data sources

The primary outcome was death from COVID-19 (ICD-10 codes U071 and U072) during the study period as recorded in the CDR. Following Rosengren et al.’s [38] definition, death from COVID-19 was considered where COVID-19 was either the underlying cause of death or a contributing diagnosis to an underlying cause of death likely to be COVID-19-related. For acceptable COVID-19-related underlying causes of death, see Appendix Table A.2. To ascertain that causes of death unrelated to COVID-19 were excluded, the underlying causes of death had to exhibit symptoms and complications (see Table A.3) that were consistent with COVID-19. Figure 2 presents the algorithm applied to determine COVID-19 deaths from the data sources. Participants were censored upon death from causes unrelated to COVID-19 or at the end of the study period. Censoring those who relocated during the study period was not possible as our data contained only one residential address per year.

### Secondary outcome

**Fig. 3:**
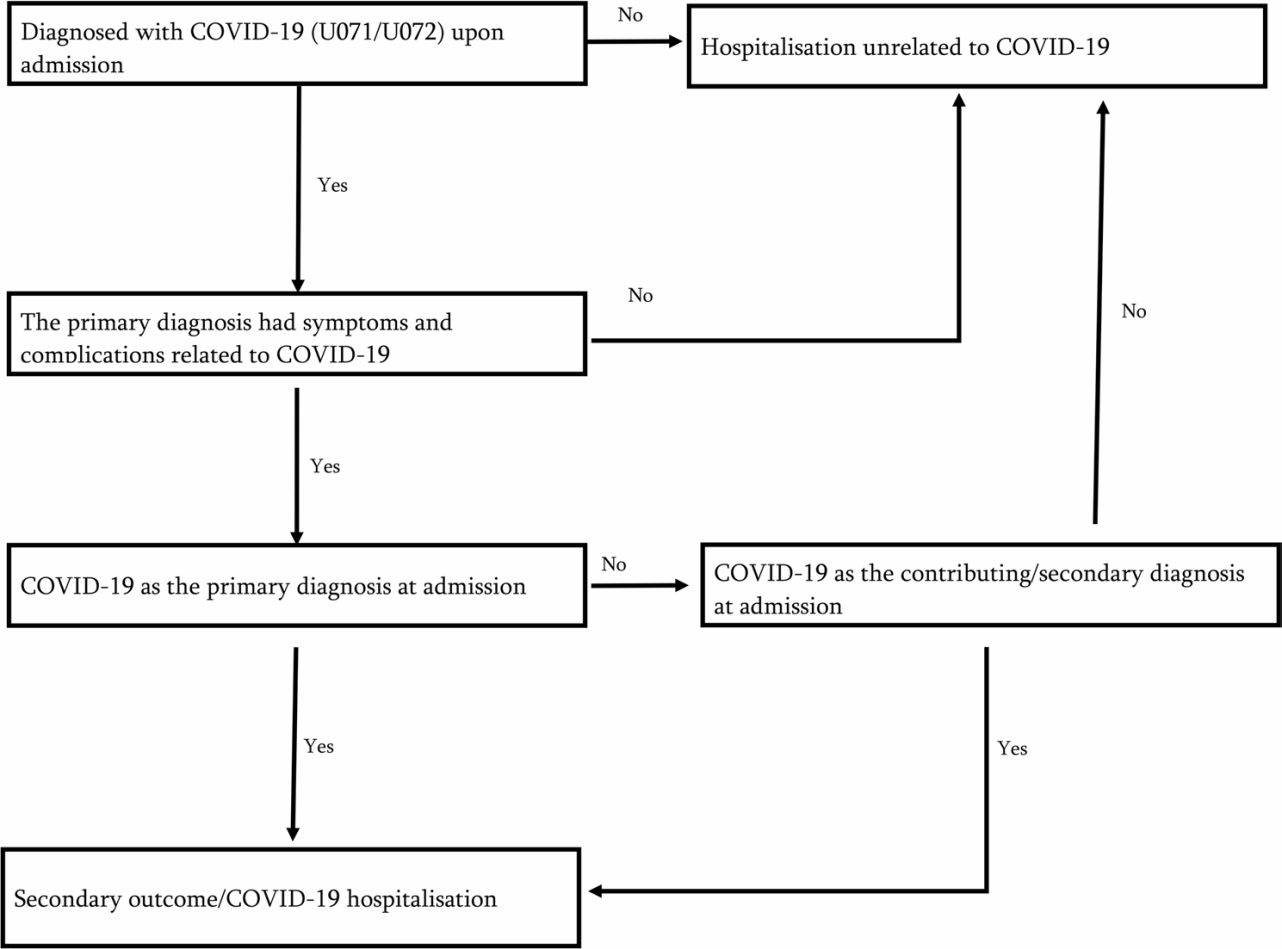
The algorithm used to determine COVID-19 hospitalisations (secondary outcome) from data sources

COVID-19 hospitalisation was also defined according to Rosengren et al. [38], as hospital admission with either COVID-19 (ICD-10 codes U071 and U072) as the primary diagnosis or contributory diagnosis to a principal diagnosis likely to be COVID-19 related, during the study period, as recorded in the NPR. Table A.3 details the list of acceptable COVID-19-related symptoms and principal diagnoses. The principal diagnoses had to present symptoms and complications that were compatible with COVID-19. Participants were censored upon death or at the end of the study period. COVID-19 hospitalisation cases were determined from the data sources through the algorithm in Fig. 3.

### Main exposure

At baseline, data on PSDs were retrieved from the ScOut database and linked to Register RELOC-AGE using geocodes of the residential address of each individual. ScOut is a recently developed data source on 24 perceived outdoor environment characteristics assessed through different population studies in Scania between 2008 and 2019 [20].

To assess each PSD, respondents of different surveys were asked whether the quality of nature outdoors could be described as serene, natural, diverse or cohesive, within 5–10 min of walking distance from their homes. The questions were phrased as follows: “Think of nature within 5–10 minutes walking distance from where you live”. “For example, this could be green spaces, parks, or forest areas. Do you agree with the following statements? Choose an option from each line! Nature in the area where I live is (a) serene – quiet one can hear nature’s own sounds; (b) natural – has nature that is wild and fascinating, (c) diverse – has a large diversity of animal and plant species; or (d) cohesive – forms a large and cohesive area that is separated from the outside world” [20]. The response to each item was graded using a Likert scale: “agree”, “agree completely”, “disagree”, “disagree completely”, “do not know/cannot say”, and “missing”. The responses “agree” and “completely agree” were considered positive assessments, indicating that the PSD was “present” in the neighbourhood, whereas “disagree”, “disagree completely”, “do not know/cannot say”, and “missing” were treated as negative assessments, indicating the PSD was “absent”. Subsequently, participants were grouped based on their geocodes into 5,472 distinct 1 km^²^ areas covering 97% of the population in Scania [20]. In each area, the proportion of positive assessments for each PSD was estimated using random effects logistic regression, adjusted for seasonality, demographic and socioeconomic characteristics [20].

For the current study, four PSDs reflecting greenness in the residential natural outdoor environment– serene, natural, diverse, and cohesive – were used. These four PSDs have been found to be moderately or strongly correlated with each other on the individual and area levels [20], and we therefore decided to use them as a combined single score. Similar to in previous publications [22, 24, 39], scores for serene, natural, diverse and cohesive were standardised (mean = 0 and standard deviation = 1) and summed to compute the Perceived Sensory Dimension Score (PSD-score). To aid in making comparisons, the PSD-score was categorised into low, intermediate and high based on tertiles.

### Demographic characteristics

Age, sex, marital status and country of birth from the TPR were selected as potential confounders based on previous systematic reviews [3, 9, 40] investigating the association between exposure to green outdoor environments and health. Age at baseline was categorised into “59–64” (reference), “65–69”, “70–74”, “75–79”, “80–84”, and “≥ 85” years. Sex was dichotomised into “male” (reference) and “female”, country of birth into “Nordic” (i.e., Denmark, Finland, Iceland, Norway, and Sweden/as reference) and “non-Nordic”, and marital status into “single” (reference) and “married/registered partnership”.

### Socioeconomic characteristics

Data on education, income, housing tenure, and household composition for 2019 were extracted from LISA [32], while population density data for 2020 were retrieved based on the residential geocodes for the entire adult population of Scania that were monitored for vaccine uptake and effectiveness [41]. These characteristics were considered potential confounders on the basis of previous studies on the health effects of green outdoor environments [3, 40] and risk factors for COVID-19 death and hospitalisation [42, 43].

Education was categorised into “primary” (reference), “secondary” and “tertiary” in accordance with the Swedish education system [44]. The “primary” group comprised individuals with ≤ 9 years of compulsory (pre-gymnasium) education, whereas the “secondary” group had completed gymnasium education, and the “tertiary” group had “professional/university or college” education.

To facilitate comparisons of economic standards across households, we used the total household disposable income per total consumption unit, using scales adapted to Swedish living conditions [45]. The total household disposable income is the total household income received minus taxes, and the total consumption units are consumption weights assigned based on the composition of each household [32]. For example, the consumption weight for one adult is 1.0 unit and that for the first child is 0.52 [46], resulting in total consumption units of 1.52 for the household.

Housing type, tenure, and housing coordinates for 2018–2019 came from the REPR [33, 47] and the Geographical database [33]. Housing tenure was defined as “rented” (reference), “tenant-occupied”, and “owner-occupied”, which aligns with the tenures in the Swedish housing sector [48]. Thus, “rented” were individuals renting homes from landlords or housing companies, and “tenant-occupied” had rights to reside based on their shares and membership in certain tenant-owner housing associations. “Owner-occupied” had full ownership rights to their homes [48]. Household composition was defined in terms of whether the individual lived with children aged < 17 years (denoted as “yes”) or not (denoted as “no”, reference).

Population density from Statistics Sweden was calculated as the number of people per ScOut area (1 km^2^) and further categorised into low (1 to 903; reference), medium (904 to 3002) and high density (3055 to 17267) based on tertiles.

### Comorbidities

Endocrine diseases, obesity, cardiovascular diseases, stroke, renal diseases, lung diseases and cancer were identified from existing literature as potential mediators, indicating that these comorbidities are associated with exposure to green outdoor environments, COVID-19 mortality and hospitalisation [7, 40, 49–51]. Although the evidence is mixed, receiving home care services can be seen as a proxy for frailty [52–54], and is hence a risk factor for severe COVID-19 [55] and is also associated with green outdoor environment exposure [56, 57].

Data on individuals who were hospitalised with the above medical conditions between 2017 and 2019 came from the NPR (for ICD-10 codes, see Appendix Table A.4). The NPR only contains data for patients who have been hospitalised or received specialist treatment. Individuals who were hospitalised or received specialist treatment at least once were categorised as “yes”, whereas the remainder were categorised as “no” (reference).

Based on the NRCS data, receiving home care services in 2019 was classified into “yes” and “no” (reference).

### Data analysis

**Fig. 4:**
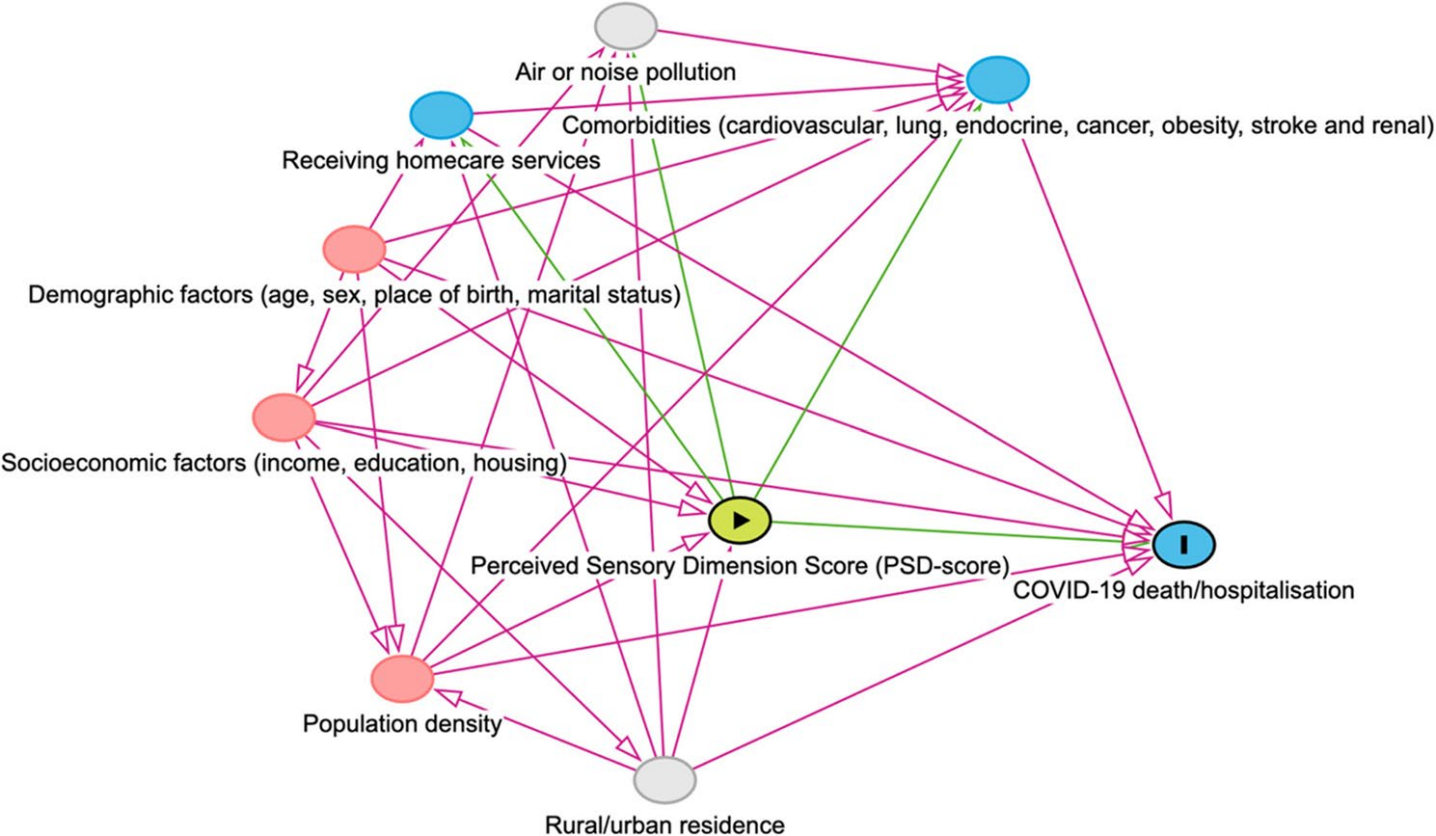
The directed acyclic graph (DAG) for the relationship between the Perceived Sensory Dimension Score (PSD-score) and COVID-19 death/hospitalisation.

Background characteristics of the study population were described by frequencies and proportions for categorical data and means and standard deviations for numeric data. The number of participants with missing data for each variable is shown in Table 1. Regression analysis was performed on complete cases only. The effect of the PSDs on each of the outcomes was assessed with Cox proportional hazards regression. Directed Acyclic Graphs (DAGs) were built using DAGitty [58] to map out the relationship between the main exposure, outcomes and covariates based on existing literature (see Fig. 4). The first regression model represented the unadjusted association between the main exposure and each of the outcomes

**Table 1:** Baseline characteristics of Register RELOC-AGE Scania residents, final sample and across perceived sensory dimension score (PSD-score) groups

Study characteristics	Register RELOC-AGE Scania residents assessed for eligibility	Final sample	Low-PSD-score^a^	Intermediate-PSD-score^b^	High-PSD-score^c^
	n (%)	n (%)	n (%)	n (%)	n (%)
Number of people	328 263	299219 (100)	99789 (33.4)	99845 (33.4)	99585 (33.3)
Age, years^d^	72 (9)	72.1 (8.8)	72.0 (9.0)	72.5 (8.9)	71.8 (8.4)
Sex					
Males Females	153993 (46.9) 174270 (53.1)	140755 (47.0) 158464 (53.0)	45462 (45.6) 54327 (54.4)	45778 (45.9) 54067 (54.2)	49515 (49.7) 50070 (50.3)
Country of birth					
Nordic Non-Nordic	286897 (87.4) 41355 (12.6)	260978 (87.2) 38233 (12.8)	77506 (77.7) 22279 (22.3)	89259 (89.4) 10586 (10.6)	94215 (94.6) 5368 (5.4)
Missing = 11					
Marital status					
Single Married/registered partner	153427 (46.7) 174836 (53.3)	136349 (45.6) 162870 (54.4)	52332 (52.4) 47457 (47.6)	46389 (46.5) 53456 (53.5)	37628 (37.8) 61957 (62.2)
Education					
Primary Secondary Tertiary	89370 (27.3) 137982 (42.2) 99529 (30.5)	79900 (26.8) 126241(42.4) 91929 (30.8)	26664 (26.9) 41046 (41.4) 31513 (31.8)	27314 (27.4) 42917 (43.1) 29322 (29.5)	25922 (26.1) 42278 (42.6) 31094 (31.3)
Missing = 1382					
Income					
Low income Middle income High income	109545 (33.4) 109335 (33.3) 109383 (33.3)	100083(33.5) 99778 (33.4) 99358 (33.2)	39729 (39.8) 31374 (31.4) 28686 (28.8)	33328 (33.4) 34373 (34.4) 32144 (32.2)	27026 (27.1) 34031 (34.2) 38528 (38.7)
Living with children					
No Yes	323496 (98.6) 4767 (1.5)	294843(98.5) 4376 (1.5)	98137 (98.3) 1652 (1.7)	98542 (98.7) 1303 (1.3)	98164 (98.6) 1421 (1.4)
Housing tenure					
Rented Tenant-owned Owner-occupied	89019 (27.1) 76314 (23.3) 162921 (49.6)	75265 (25.2) 71752 (24.0) 152193(50.9)	35464 (35.5) 36049 (36.1) 28274 (28.3)	29376 (29.4) 25836 (25.9) 44629 (44.7)	10425 (10.5) 9867 (9.9) 79290 (79.6)
Missing = 9					
Population density					
Low Medium High	109467 (33.4) 109412 (33.3) 109377 (33.3)	95351 (31.9) 101891(34.1) 101972(34.1)	4083 (4.1) 25579 (25.6) 70127 (70.3)	20841 (20.9) 48231 (48.3) 30773 (30.8)	70427 (70.7) 28081 (28.2) 1072 (1.1)
Missing = 7					
Receiving homecare					
No Yes	286003 (87.1) 42260 (12.9)	266106 (88.9) 33113 (11.1)	87330 (87.5) 12459 (12.5)	87824 (88.0) 12021 (12.0)	90952 (91.3) 8633 (8.7)
Cardiovascular diseases					
No Yes	319410 (97.3) 8853 (2.7)	291416 (97.4) 7803 (2.6)	97107 (97.3) 2682 (2.7)	97077 (97.2) 2768 (2.8)	97232 (97.6) 2353 (2.4)
Lung diseases					
No Yes	324386 (98.8) 3877 (1.2)	295821 (98.9) 3398 (1.1)	98452 (98.7) 1337 (1.3)	98704 (98.9) 1141 (1.2)	98665 (99.1) 920 (0.9)
Renal diseases					
No Yes	325014 (99.0) 3249 (1)	296364 (99.1) 2855 (1.0)	98755 (99.0) 1034 (1.0)	98869 (99.0) 976 (1.0)	98740 (99.2) 845 (0.9)
Endocrine diseases					
No Yes	323147 (98.4) 5116 (1.6)	294740 (98.5) 4479 (1.5)	98141 (98.4) 1648 (1.7)	98365 (98.5) 1480 (1.5)	98234 (98.6) 1351 (1.4)
Stroke					
No Yes	327120 (99.7) 1143 (0.4)	298296 (99.7) 923 (0.3)	99480 (99.7) 309 (0.3)	99520 (99.7) 325 (0.3)	99296 (99.7) 289 (0.3)
Obesity					
No Yes	327702 (99.8) 561 (0.2)	298725 (99.8) 494 (0.2)	99616 (99.8) 173 (0.2)	99699 (99.9) 146 (0.2)	99410 (99.8) 175 (0.2)
Cancer					
No Yes	324313 (98.8) 3950 (1.2)	295650 (98.8) 3569(1.2)	98595 (98.8) 1194 (1.2)	98604 (98.8) 1241 (1.2)	98451 (98.9) 1134 (1.1)

^a^Low-Perceived Sensory Dimension Score: median (minimum to maximum) = −3.8 (−6.5 to −2.3)

^b^Intermediate-Perceived Sensory Dimension Score: median (minimum to maximum) = −0.7 (−2.3 to 1.5)

^c^High-Perceived Sensory Dimension Score: median (minimum to maximum) = 4.2 (1.5 to 11.0)

^d^Data are presented as mean (standard deviation)

In the second regression model, demographic and socioeconomic characteristics were adjusted for. Further, adjusting for population density, an area-level variable, in the third regression model isolated the effect that might be attributed to the PSD-score on COVID-19 death or hospitalisation, net of population density. In addition, a direct effect of PSD-score on the outcomes was estimated in a fourth model by adding mediators, including comorbidities and home care services, in the fourth regression model. Confidence intervals (CI) were established at 95%. The likelihood ratio test was applied to examine whether any of the exposure categories exhibited an impact on the outcome. Effect modification was assessed using interaction terms between PSD-score and income/education in Cox regression, and a p-value < 0.05 was considered statistically significant.

The assumption of proportional hazards was tested by including interaction terms between covariates and the logarithm of survival time in the Cox regression model. As the assumption of proportional hazards was violated in the final models, sensitivity analysis was conducted to show the extent to which the hazard ratios varied with time. This involved splitting the study period (1 January to 31 December 2020) into two, using the median time point where 50% of COVID-19 deaths/hospitalisations had occurred. This was 19 November 2020 for COVID-19 death, and 20 November 2020 for COVID-19 hospitalisation. Data were analysed using STATA/SE version 17.0 (StataCorp, College Station, USA).

## Results

The baseline characteristics between the source population and the sample were comparable for all variables included in the models. The mean age in the study was 72 years, with a higher proportion of women (*N* = 158,464; 53%). Population density was inversely associated with the PSD-score. Among individuals in the low PSD-score tertile, most individuals (70.3%) belonged to the high population density tertile, whereas in the high PSD-score tertile, most (70.7%) were in the low population density tertile. Nordic origin, being married/registered partner, higher income and education, as well as home ownership, were linked to living in higher PSD-score areas. For further details, see Table 1.

A total of 426 COVID-19 deaths were recorded over the 295,670 person-years of follow-up. COVID-19 was the underlying cause of death in 418 cases and a contributing diagnosis to a likely COVID-19-related underlying cause in 8. Thus, the COVID-19 death rate was 14.4 per 10,000 person-years. The highest death rate per 10,000 person-years was in the low- (18.9), followed by the intermediate (14.5), and high PSD-score (9.8) groups. Table 2 details the COVID-19 death rates across the PSD-score categories.

**Table 2:** COVID-19 death rate among study participants according to the perceived sensory dimension score (PSD-score)

	Study population	Low PSD-score	Intermediate PSD-score	High PSD-score
Number of deaths	426	186	143	97
Person years at risk	295,670	98497.8	98581.5	98590.7
Death rate/10,000 person-years (95% CI)	14.4 (13.1–15.8)	18.9 (16.4–21.8)	14.5 (12.3–17.1)	9.8 (8.1–12.0)

There were 1417 COVID-19 hospitalisations during the 295,397 person-years of follow-up. At admission, COVID-19 was the primary diagnosis in 1,376 cases and a contributing diagnosis in 41 primary diagnoses likely to be COVID-19-related. Accordingly, the COVID-19 hospitalisation rate per 10,000 person-years was 48. The low PSD-score group had the highest hospitalisation rate of 68.9 per 10,000 person-years, followed by the intermediate PSD-score group (43.5 per 10,000), with the high PSD-score group recording the lowest (31.5 per 10,000 person-years). For more details, see Table 3.

**Table 3:** COVID-19 hospitalisation rates among study participants according to the perceived sensory dimension score (PSD-score)

	Study population	Low PSD-score	Intermediate PSD-score	High PSD-score
Number of Hospitalisations	1417	678	429	310
Person years at risk	295396.9	98357.3	98510.0	98529.6
Hospitalisation rate/10 000 person-years (95% CI)	48.0 (45.5–50.5)	68.9 (63.9–74.3)	43.5 (39.6–47.9)	31.5 (28.1–35.2)

Models I – IV in Table 4 provide the hazard ratios and their 95% CIs for the associations between PSD-score and the risk of COVID-19 death. In the unadjusted analysis (Model I), residents in intermediate (HR = 0.77; 95% CI: 0.62–0.96) and high PSD-score (HR = 0.52; 95% CI: 0.41–0.67) areas had a lower risk of COVID-19 death than their counterparts in low PSD-score areas. Having accounted for individual-level demographic and socioeconomic characteristics in Model II, the hazard ratio for COVID-19 death remained the same for residents in intermediate PSD-score areas (HR = 0.77; 95% CI: 0.61–0.96) but increased to 0.67 (95% CI: 0.51–0.88) for those in high PSD-score areas. Upon adding population density in Model III, the protective effects against COVID-19 death attenuated to 0.86 in both low and intermediate PSD-score groups, and became not statistically significant. Further adjusting for comorbidities weakened the association between COVID-19 death and the intermediate (HR = 0.90; 95% CI: 0.71–1.15) and high PSD-score groups (HR = 0.88, 95% CI: 0.62–1.24). The likelihood ratio test comparing the fully adjusted models, with and without PSD-score, was not statistically significant (*p* = 0.66). This showed that evidence for the association between PSD-score and COVID-19 death was inconclusive. Table A.5 in the Appendix provides the full models for the hazard ratios and their 95% CIs for the associations between the PSD-score and the risk of COVID-19 death.

**Table 4:** Cox regression models for the associations between perceived sensory dimension score (PSD-score) and COVID-19 death

Exposure variables	Model I^a^ (95% CI)^e^ *N* = 299,156	Model II^b^ (95% CI) *N* = 297,990	Model III^c^ (95% CI) *N* = 297,985	Model IV^d^ (95% CI) *N* = 297,985
PSD-score				
Low-PSD-score Intermediate-PSD-score High-PSD-score	Reference 0.77 (0.62–0.96) 0.52 (0.41–0.67)	Reference 0.77 (0.61–0.96) 0.67 (0.51–0.88)	Reference 0.86 (0.68–1.10) 0.86 (0.61–1.21)	Reference 0.90 (0.71–1.15) 0.88 (0.62–1.24)

Analysis in this table covers the study period from 1 January to 31 December 2020

^a^Model I: Unadjusted

^b^Model II: adjusted for age, sex, marital status, country of birth, education, income and housing tenure

^c^Model III: adjusted for age, sex, marital status, country of birth, education, income, housing tenure and population density

^d^Model IV: adjusted for variables in Model III plus cancer, cardiovascular diseases, endocrine diseases, renal diseases, stroke, obesity, lung diseases and receiving homecare services

^e^95% CI: 95% Confidence Interval

Table 5 presents the summarised Models I – IV, including the hazard ratios and their 95% CIs for the association between PSD-score and the risk of COVID-19 hospitalisation.

**Table 5:** Cox regression models for the associations between perceived sensory dimension score (PSD-score) and COVID-19 hospitalisation

Exposure variables	Model I^a^ (95% CI)^e^ *N* = 299,156	Model II^b^ (95% CI) *N* = 297,990	Model III^c^ (95% CI) *N* = 297,985	Model IV^d^ (95% CI) *N* = 297,985
PSD-score				
Low-PSD-score Intermediate-PSD-score High-PSD-score	Reference 0.63 (0.56–0.71) 0.46 (0.40–0.52)	Reference 0.74 (0.65–0.84) 0.69 (0.59–0.80)	Reference 0.85 (0.75–0.98) 0.90 (0.74–1.09)	Reference 0.88 (0.77–1.01) 0.92 (0.76–1.12)

This analysis included community-dwellers aged 59 years or above during the study period 1 January to 31 December 2020 in Scania

^a^Model I: Unadjusted

^b^Model II: adjusted for age, sex, marital status, country of birth, education, income, housing tenure and living with children

^c^Model III: adjusted for age, sex, marital status, country of birth, education, income, housing tenure, living with children and population density

^d^Model IV: adjusted for variables in Model III plus cancer, cardiovascular diseases, endocrine diseases, renal diseases, stroke, obesity, lung diseases and receiving homecare services

^e^95% CI: 95% Confidence Interval

The full models can be found in Table A.6 in the Appendix. In the unadjusted Model I, the hazard ratios for COVID-19 hospitalisation were 0.63 (95% CI: 0.56–0.71) in the intermediate and 0.46 (95% CI: 0.40–0.52) in the high PSD-score group, compared to the low PSD-score group. Adjusting for individual-level demographic and socioeconomic factors in Model II weakened the association with COVID-19 hospitalisation, resulting in a hazard ratio of 0.74 (0.65–0.84) and 0.69 (95% CI: (0.59–0.80) for the intermediate- and high-PSD-score residents, respectively. Further adjustment in Model III for population density increased the point estimates towards the null value, with the hazard ratios at 0.85 (95% CI: 0.75–0.98) in the intermediate and 0.90 (95% CI: 0.74–1.09) in the high PSD-score group. As shown in Model IV, adding comorbidities further attenuated the protective effects, with the COVID-19 hospitalisation hazard ratios at 0.88 (95% CI: 0.77–1.01) and 0.92 (95% CI: 0.76–1.12) in the intermediate and high PSD-score groups, respectively. The likelihood ratio test comparing Model IV with and without PSD-score, was not statistically significant (*p* = 0.17), highlighting insufficient evidence for the association between PSD-score and the risk of COVID-19 hospitalisation.

The only statistically significant interaction in relation to the hazard of COVID-19 death (Table A.7) was between intermediate PSD-score and tertiary education (*p* = 0.03). The likelihood ratio test comparing Model IV (Table A.7), with and without interaction terms, however, showed that effect moderation by education in the association between PSD-score and the hazard of COVID-19 death was not statistically significant. There was insufficient evidence to indicate that the association between PSD-score and COVID-19 death risk varied according to income (Table A.8). Similarly, the association between PSD-score and COVID-19 hospitalisation risk did not significantly differ according to education and income (see Table A.9 and Table A.10).

The hazard ratios for the association between PSD-score and the risk of COVID-19 death and hospitalisation before and after the median time points are presented in Table A.11 and Table A.12 and Table A.13 and Table A.14. The hazard ratios between these time points were not statistically significant.

Due to the strong effect of the adjustment for population density on the estimated effect of the PSD-score, a post-hoc analysis was conducted to investigate differences in COVID-19-related death and hospitalisation risk stratified by population density (see Table A.15). The results indicated effect modification by population density in the association between PSD-score and COVID-19-related death. An intermediate or high PSD-score was not significantly associated with lower COVID-19-related death risk in low and medium-density population areas, but a significantly higher risk of COVID-19 death (HR 3.07; 95% CI: 1.10–8.56) was seen for the high PSD-score group in high-density residential areas. Similar (statistically not significant) patterns were observed for COVID-19-related hospitalisation (see Table A.16).

## Discussion

This one-year longitudinal study during the pre-vaccination period of the COVID-19 pandemic set out to investigate the association between residential natural outdoor environment (indicating greenness) and the risk of COVID-19-related death or hospitalisation, and whether these associations differed by income and education among community-dwelling adults aged 59 years and older in Scania, Sweden. Although the unadjusted models indicated that exposure to residential natural outdoor environments reduced the risk of COVID-19-related death and hospitalisation, these associations became weaker upon adjustment for population density. Moreover, we found no evidence that the associations between PSD-score and COVID-19 death or hospitalisation were moderated by income and education.

The mechanisms through which exposure to green outdoor environments supports health remain poorly understood in the literature [59, 60]. In the current study, we hypothesised that exposure to residential natural outdoor environment, measured as PSDs reflecting greenness, influenced COVID-19 deaths and hospitalisations. We further hypothesised that PSD-score might indirectly influence COVID-19 outcomes through known effects on cardiovascular diseases, renal diseases, obesity, endocrine diseases, cancer, lung diseases and stroke [3, 4, 7, 40, 49, 50], and via other, “direct,” pathways. We found no substantial differences in the hazards of COVID-19 death or hospitalisation between the direct and total effect models, suggesting that mediating effects by comorbidities might be absent.

However, data on comorbidities in the current study were based on records of individuals who had been admitted to a hospital in Sweden or received specialist consultation between 2017 and 2019, thus capturing only the most severe conditions. Further, green outdoor environments influence health through multiple pathways [4], so other environmental factors (e.g., urban/rural residence, air pollution, and noise [4, 38]) might be involved in the association between greenness and COVID-19 outcomes. Taken together, the mechanisms through which green outdoor environments relate to COVID-19 deaths/hospitalisations warrant further exploration, which should also consider additional environmental exposures.

In the unadjusted models and those adjusted for demographic and socioeconomic characteristics only, there was an apparent dose-response pattern such that the protective effects of residential natural outdoor environments strengthened as the PSD-score increased from intermediate to high PSD-score. Thus, the effect sizes in the intermediate PSD-score group were closer to the null value than those in the high PSD-score groups. This gradient, however, was less evident when population density was added into the models, as the effect sizes across the intermediate and high PSD-score categories became almost comparable.

Moreover, accounting for population density weakened the association between the PSD-score and COVID-19-related death or hospitalisation and caused a loss of statistical precision. Overall, the current study did not find firm evidence for the association between residential natural outdoor environments measured through PSDs and COVID-19-related death and hospitalisation in the fully adjusted models. However, it was observed that high population density was strongly associated with a higher hazard ratio for COVID-19 death and hospitalisation, more so during the period from November to December 2020 (the second wave of the COVID-19 pandemic) in Scania.

In Stockholm, on the other hand, population density has been strongly linked to COVID-19 death and hospitalisation during the first wave of the COVID-19 pandemic [51]. Although population density was inversely associated with PSD-score in the current study, it appears to have a more pronounced negative impact on COVID-19 death/hospitalisation rates than low PSD-scores. Moreover, post-hoc analytic findings revealed that population density was also an effect modifier, as a high PSD-score in high-density residential areas increased the risk for COVID-19 death/hospitalisation. This was in contrast to areas with low and medium population density, in which residential natural outdoor environment exposure had a potentially protective effect. This finding was unexpected, and we do not have a firm explanation for it. There is some evidence that mobility around parks and greenness surrounding residential areas were related to increased COVID-19 cases and related mortality [10, 61]. Thus, one possibility could be that natural outdoor environments in high-density residential areas might contribute to more gatherings or crowding, potentially leading to COVID-19-related deaths and hospitalisations [61].

In general, the findings of the current study align with an ecological study in the U.S. that also reported no statistically significant associations between greenness and COVID-19 mortality at the national level [62], having adjusted for confounders, including population density. This contrasts with the results of an ecological study by Russette et al., [63] in which exposure to greenness was linked to a lower risk of COVID-19 death in a dose-response pattern. In addition, a Danish cohort study [12] found that greenness was associated with a lower risk of COVID-19 death and hospitalisation. Differences in findings might be explained by different study designs as well as greenness measurements. For example, Russette et al., [63] adjusted for total population per county instead of population density, while in the Danish [12] study, the NDVI was used to measure the quantity of greenness with adjustment for population density. The point estimates obtained in our study suggest that the protective effects of residential outdoor environment exposure on COVID-19-related hospitalisation are comparable to those documented in studies focusing on the associations between greenness and non-communicable diseases. For instance, several studies in a systematic review [64] found that exposure to greenness provided similar protection from hospital admission due to cancer, cardiovascular diseases and mental health.

Although the evidence is mixed [11], other studies found that the health benefits of green outdoor environments varied according to education and income levels [12, 28]. In contrast, the current study did not find evidence for effect modification.

Literature on how perceived greenness influences the risk of COVID-19 death or hospitalisation is scarce. Previous publications looked into the association between perceived greenness and self-reported physical health. For example, a survey in Australia found that perceived neighbourhood greenness increased the odds of better self-reported physical and mental health [65].

Moreover, a cross-sectional study in China reported similar findings, where perceived greenness was positively associated with better self-reported physical health, more so in larger cities [66]. In contrast, a cross-sectional study [67] using in-person administered questionnaires in Beijing found no significant correlation between perceived greenness and physical health. The use of individual self-reports to assess perceived outdoor greenness may cause bias if these reports are related to individual-level factors that influence the outcome. To counteract such bias, we used the aggregated area-level PSD-score [22] available through the ScOut database [20], which is considered a compromise measure between perceived and objectively measured greenness in the residential outdoor environment. Further, due to the moderate-to-high correlation of these four PSDs demonstrated previously [20], a single PSD-score was calculated in the current study to measure the exposure.

### Strengths and limitations

Previous research on the health effects of greenness has mainly focused on non-communicable diseases (e.g., cancer, diabetes, and hypertension) [3, 4, 9, 64]. To the best of our knowledge, this is the first study utilising individual-level data to provide insights into how perceived qualities of green outdoor environments influence communicable diseases such as COVID-19. Another strength of our research is that we utilised high-quality Swedish population registers with well-defined variables and little missing data (less than 0.5% highest missing). In addition, the comparable baseline characteristics between the source population and the sample suggest a low risk of selection bias and the possibility of generalising the findings to a broader population of older adults in Sweden. Next, we adopted a longitudinal study design with exposure measured before the outcome occurrence and a separation between the total and direct effects of the PSD-score.

Although we were able to account for several potential confounders, residual confounding due to unobserved variables (e.g., rural/urban residency) remains a possible study limitation. To ensure participants were exposed to the same area for at least two years, individuals who had relocated at least once within the two years preceding the study period were excluded. However, tracking whether participants relocated multiple times during the same year was not possible, as there is only one housing record available per year in the registers. This might have resulted in exposure misclassification bias, provided that some participants might have moved from their baseline areas to different residential locations during the year. However, yearly relocation rates among older adults are low (approximately 5%) according to Statistics Sweden. Hence, major bias due to exposure misclassification is not plausible.

## Conclusions

Residential natural outdoor environment was associated with a lower risk of COVID-19-related death and hospitalisation in the unadjusted and partially adjusted models, although these associations became weaker and statistically imprecise upon adjustment for population density. Our post-hoc analysis indicated that the observed associations between PSD-score and COVID-19 outcomes might be attributable to differences in population density across different levels of exposure. No moderating effects by education and income were detected.

Future studies utilising longitudinal study designs across diverse populations may provide further insight into the interaction between the natural outdoor environment and population density in the context of infectious diseases such as COVID-19. Taken together, the unfolding climate crisis, urban densification, and the threat of future epidemics and pandemics represent significant public health challenges, especially for older adults. The findings of the current study underscore the need for age-friendly living environments that can help buffer the negative impact of these emerging threats.

## Supplementary Information

Supplementary Material 1

## Data Availability

ScOut data management and analysis codes are available upon request. Sharing individual-level data requires new ethical approval and a confidentiality review by Lund University.

## Abbreviations

PSDs: Perceived Sensory Dimensions
PSD-score: Perceived Sensory Dimension Score
TPR: Total Population Register
LISA: Longitudinal Integrated database for Health Insurance and Labor Market Studies
PEPR: Real Estate Property Register
AR: Apartment Registers
ScOut: Scania outdoor environment database
NPR: National Patient Register
DR: Cause of Death Register
NRCS: National Register of Care and Social Services for the Elderly and Persons with Impairments
ICD-10: 10th revision of the International Classification of Diseases
DAGs: Directed Acyclic Graphs
CI: Confidence Interval
