# Supplementary material for: The role of natural outdoor environment on COVID-19 mortality and hospitalisations among older community-dwellers in the pre-vaccination period: the Register RELOC-AGE cohort study in Sweden
Source: BMC Public Health. 2025 Dec 7;26:238. doi: 10.1186/s12889-025-25515-w (PMC12821256; doi:10.1186/s12889-025-25515-w)
Supplement: Supplementary file 1 — Supplementary Material 1 [file 12889_2025_25515_MOESM1_ESM.docx]

# Appendix

**Table A.1: Characteristics of the data sources, within Register RELOC-AGE, used in this study.**

| **Data source** | **Dataset timeline** | **Data holder** | **Study variables** |
| --- | --- | --- | --- |
| Total Population Register (TPR) | 1990–2020 | Statistics Sweden | Sex, age, marital status, and country of birth |
| Longitudinal Integrated database for Health Insurance and Labor Market Studies (LISA) | 1990–2020 | Statistics Sweden | Education, household composition, and income |
| Real Estate Property and Apartment Register | 1990–2020 | Statistics Sweden | Housing tenure and type |
| Geographical database | 1990–2020 | Statistics Sweden | Housing coordinates |
| Scania outdoor environment database (ScOut) | 2008–2019 | Lund University Population Data Platform (LUPOP) | Perceived greenness: serene, natural, diverse, and cohesive |
| National Patient Register (NPR) | 1987–2021 | National Board of Health and Welfare | COVID-19-related hospitalisations, and comorbidities |
| Death Cause Register (DCR) | 1990–2021 | National Board of Health and Welfare | COVID-19-related deaths |
| National Register of Care and Social Services for the Elderly and Persons with Impairments (NRCS) | 2020 | National Board of Health and Welfare | Receiving homecare services |
| Statistics Sweden | 2020 | Statistics Sweden | Population density |

**Table A.2: COVID-19-related underlying causes of death from the Cause of Death Register.**

| **Underlying cause of death** | **ICD-10 codes^a^** |
| --- | --- |
| Lung diseases | B99, J96, J44, J45 |
| Cardiac diseases | I25, I30, I48, I50 |
| Renal conditions | N17, N18, N19, E87 |

^a^ICD-10 codes: Tenth Revision of the International Classification of Diseases.

For a cause of death to be a COVID-19 death, the underlying cause of death had to exhibit symptoms and complications that are compatible with COVID-19.

Table adapted from Rosengren et al., [39].

**Table A.3: COVID-19-related symptoms and principal diagnosis extracted from the Swedish National Patient Register.**

| **Primary diagnosis** | **ICD-10 codes^a^** |
| --- | --- |
| COVID-19 symptoms^b^ | R05, R06, R07, R09, R42, R50, R51, R53, R55 |
| Lung diseases | J00, J03, J06, J10, J128, J129, J13, J15, J168, J17, J18, J22, B342, B349, B972, B99, J26, J81, J80, J90, J96, J99, J208, J209, J218, J219, J44, J45 |
| Cardiac diseases | B332, I25, I30, 132, I40, I41, I48, I50, R00 |
| Renal conditions | N17, N18, N19, B87 |

^a^ICD-10 codes: Tenth Revision of the International Classification of Diseases.

^b^COVID-19 symptoms: the principal diagnosis had to present these symptoms that are consistent with COVID-19 for it to be considered a COVID-19 hospitalisation.

Table adapted from Rosengren et al., [39].

**Table A.4: ICD-10 codes^a^ for comorbidities of study participants as recorded in the Swedish National Patient Register.**

| **Comorbidities** | **ICD-10 codes** |
| --- | --- |
| Cardiovascular diseases | J00, J03, J06, J10, J128, J129, J13, J15, J168, J17, J18, J22, B342, B349, B972, B99, J26, J81, J80, J90, J96, J99, J208, J209, J218, J219, J44, J45 |
| Lung diseases | B332, I25, I30, 132, I40, I41, I48, I50, R00 |
| Endocrine diseases | N17, N18, N19, B87 |
| Stroke | G93, I676, I677, I679, I69, I690, I690A, I690B, I691, I693, I694 |
| Renal diseases | M103, N00-N08, N10-N16, N17-N19, N200, N289 |
| Obesity | E660, E661, E662, E668, E669 |
| Cancer | C100-C14, C30-C39, C40-C41, CC43-C44, C45-C49, C50-C50, C51-C58, C60-C63, C64-C68, C69-C72, C73-C75, C76-C80, C81-C96, C97-C97 |

^a^ICD-10 codes: Tenth Revision of the International Classification of Diseases

**Table A.5: Cox regression models for the associations between Perceived Sensory Dimension Score (PSD-score) and COVID-19 death.**

| **Exposure variables** | **Model I (95% CI)^a^**  **N = 299156** | **Model II (95% CI)**  **N = 297990** | **Model III (95% CI)**  **N = 297985** | **Model IV (95% CI)**  **N = 297985** |
| --- | --- | --- | --- | --- |
| **PSD-score**  Low-PSD-score  Intermediate-PSD-score  High-PSD-score | Reference  0.77 (0.62 - 0.96)  0.52 (0.41 - 0.67) | Reference  0.77 (0.61 - 0.96)  0.67 (0.51 - 0.88) | Reference  0.86 (0.68 - 1.10)  0.86 (0.61 - 1.21) | Reference  0.90 (0.71 - 1.15)  0.88 (0.62 - 1.24) |
| **Age categories, years**  59 – 64  65 – 69  70 – 74  75 – 79  80 – 84  85 or above |  | Reference  2.83(1.23 - 6.50)  5.22 (2.41 - 11.30)  8.67 (4.08 - 18.42)  25.29 (12.16 - 52.61)  62.40 (30.34 - 128.36) | Reference  2.82 (1.23 - 6.49)  5.21 (2.41 – 11.28)  8.64 (4.07 - 18.35)  25.16 (12.09 - 52.32)  61.74 (30.01- 127.00) | Reference  2.53 (1.10 - 5.83)  4.09 (1.89 - 8.87)  5.29 (2.48 – 11.28)  11.25 (5.36 - 23.61)  17.36 (8.30 - 36.33) |
| **Sex**  Male  Female |  | Reference  0.41 (0.33 - 0.50) | Reference  0.40 (0.33 - 0.50) | Reference  0.43 (0.35 - 0.53) |
| **Country of birth**  Nordic  Non-Nordic |  | Reference  1.18 (0.86 - 1.62) | Reference  1.15 (0.84 - 1.57) | Reference  1.24 (0.91 - 1.71) |
| **Marital status**  Single  Married/registered partnership |  | Reference  0.91 (0.74 - 1.14) | Reference  0.92 (0.74 - 1.14) | Reference  1.27 (1.01 - 1.58) |
| **Education**  Primary  Secondary  Tertiary |  | Reference  0.83 (0.67 - 1.02)  0.65 (0.49 - 0.88) | Reference  0.82 (0.66 - 1.01)  0.64 (0.48 - 0.85) | Reference  0.85 (0.68 - 1.05)  0.69 (0.52 - 0.92) |
| **Income**  Low income  Middle income  High income |  | Reference  0.87 (0.69 - 1.10)  0.79 (0.55 - 1.13) | Reference  0.87 (0.68 - 1.09)  0.77 (0.54 - 1.10) | Reference  0.95 (0.75 - 1.20)  0.91 (0.64 - 1.30) |
| **Housing tenure**  Rented  Tenant-owned  Owner-occupied |  | Reference  0.97 (0.77 - 1.23)  0.69 (0.53 - 0.89) | Reference  0.98 (0.77 - 1.23)  0.76 (0.58 – 1.00) | Reference  1.11 (0.88 - 1.41)  1.01 (0.77 - 1.33) |
| **Population density**  Low  Medium  High |  |  | Reference  1.14 (0.84 – 1.56)  1.52 (1.05 – 2.20) | Reference  1.16 (0.85 – 1.58)  1.61 (1.11 – 2.33) |
| **Receiving homecare**  No  Yes |  |  |  | Reference  6.83 (5.29 – 8.84) |
| **Cardiovascular diseases**  No  Yes |  |  |  | Reference  1.86 (1.39 - 2.49) |
| **Lung diseases**  No  Yes |  |  |  | Reference  1.78 (1.27 - 2.48) |
| **Endocrine diseases**  No  Yes |  |  |  | Reference  2.53 (1.86 - 3.44) |
| **Renal diseases**  No  Yes |  |  |  | Reference  1.50 (1.06 - 2.13) |
| **Stroke**  No  Yes |  |  |  | Reference  1.99 (1.28 - 3.10) |
| **Obesity**  No  Yes |  |  |  | Reference  1.50 (0.69 - 3.27) |
| **Cancer**  No  Yes |  |  |  | Reference  1.57 (1.09 - 2.27) |

Analysis in this table covers the study period from 1 January to 31 December 2020.

^a^95% CI: 95% Confidence Interval.

**Table A.6: Cox regression models for the associations between Perceived Sensory Dimension Score (PSD-score) and COVID-19 hospitalisation.**

| **Exposure variables** | **Model I (95% CI)^a^**  **N = 299156** | **Model II (95% CI)**  **N = 297990** | **Model III (95% CI)**  **N = 297985** | **Model IV (95% CI)**  **N = 297985** |
| --- | --- | --- | --- | --- |
| **PSD-score**  Low-PSD-score  Intermediate-PSD-score  High-PSD-score | Reference  0.63 (0.56 - 0.71)  0.46 (0.40 - 0.52) | Reference  0.74 (0.65 - 0.84)  0.69 (0.59 - 0.80) | Reference  0.85 (0.75 - 0.98)  0.90 (0.74 - 1.09) | Reference  0.88 (0.77 - 1.01)  0.92 (0.76 - 1.12) |
| **Age categories, years**  59 – 64  65 – 69  70 – 74  75 – 79  80 – 84  85 or above |  | Reference  1.22 (1.01 - 1.49)  1.20 (0.98 - 1.47)  1.81 (1.49 - 2.20)  2.88 (2.36 - 3.50)  4.59 (3.79 - 5.55) | Reference  1.22 (1.01 - 1.48)  1.20 (0.98 - 1.46)  1.81 (1.49 - 2.20)  2.87 (2.36 - 3.50)  4.56 (3.77 - 5.52) | Reference  1.16 (0.95 - 1.40)  1.05 (0.86 - 1.29)  1.39 (1.14 - 1.69)  1.82 (1.49 - 2.23)  2.15 (1.74 - 2.65) |
| **Sex**  Males  Females |  | Reference  0.54 (0.48 - 0.60) | Reference  0.54 (0.48 - 0.60) | Reference  0.59 (0.52 - 0.66) |
| **Country of birth**  Nordic  Non-Nordic |  | Reference  2.47 (2.17 - 2.82) | Reference  2.38 (2.09 - 2.72) | Reference  2.48 (2.18 - 2.83) |
| **Marital status**  Single  Married/registered partnership |  | Reference  1.02 (0.91 - 1.15) | Reference  1.03 (0.91 - 1.16) | Reference  1.16 (1.03 - 1.31) |
| **Education level**  Primary  Secondary  Tertiary |  | Reference  0.91 (0.81 - 1.03)  0.72 (0.62 - 0.84) | Reference  0.90 (0.80 - 1.02)  0.70 (0.60 - 0.81) | Reference  0.95 (0.84 - 1.07)  0.75 (0.65 - 0.88) |
| **Income level**  Low income  Middle income  High income |  | Reference  0.90 (0.79 - 1.03)  0.85 (0.71 - 1.00) | Reference  0.90 (0.79 - 1.03)  0.83 (0.70 - 0.99) | Reference  0.99 (0.87 - 1.13)  0.95 (0.80 - 1.13) |
| **Housing tenure**  Rented  Tenant-owned  Owner-occupied |  | Reference  0.75 (0.66 - 0.86)  0.60 (0.52 - 0.70) | Reference  0.76 (0.67 - 0.87)  0.69 (0.59 - 0.80) | Reference  0.83 (0.72 - 0.95)  0.80 (0.69 - 0.93) |
| **Living with children**  No  Yes |  | Reference  2.70 (2.01 - 3.61) | Reference  2.69 (2.01 - 3.59) | Reference  2.94 (2.20 - 3.94) |
| **Population density**  Low  Medium  High |  |  | Reference  1.05 (0.88 - 1.25)  1.57 (1.27 – 1.93) | Reference  1.07 (0.89 – 1.27)  1.63 (1.32 – 2.00) |
| **Receiving homecare**  No  Yes |  |  |  | Reference  2.80 (2.42 – 3.23) |
| **Cardiovascular diseases**  No  Yes |  |  |  | Reference  2.57 (2.12 - 3.10) |
| **Lung diseases**  No  Yes |  |  |  | Reference  2.08 (1.69 - 2.57) |
| **Endocrine diseases**  No  Yes |  |  |  | Reference  2.14 (1.75 - 2.63) |
| **Renal diseases**  No  Yes |  |  |  | Reference  1.49 (1.19 - 1.87) |
| **Stroke**  No  Yes |  |  |  | Reference  1.17 (0.82 - 1.67) |
| **Obesity**  No  Yes |  |  |  | Reference  1.77 (1.17 - 2.69) |
| **Cancer**  No  Yes |  |  |  | Reference  1.65 (1.31 - 2.09) |

This analysis included community-dwellers aged 59 years or above during the study period, 1 January to 31 December 2020, in Scania

^a^95% CI: 95% Confidence Interval.

**Table A.7: The Cox regression model for effect moderation by education in the association between Perceived Sensory Dimension Score (PSD-score) and COVID-19 death among study participants during the study period from 1 January to 31 December 2020**

| **Exposure variables** | **Model I (95% CI)^a^**  **N = 298007** | **Model II (95% CI)**  **N = 297990** | **Model III (95% CI)**  **N = 297985** | **Model IV (95% CI)**  **N = 297985** |
| --- | --- | --- | --- | --- |
| **PSD-score**  Low-PSD-score  Intermediate-PSD-score  High-PSD-score | Reference  0.70 (0.50 - 0.96)  0.52 (0.36 - 0.75) | Reference  0.68 (0.49 - 0.95)  0.64 (0.44 - 0.93) | Reference  0.77 (0.55 - 1.09)  0.81 (0.53 - 1.25) | Reference  0.81 (0.57 - 1.14)  0.85 (0.55 - 1.31) |
| **Education**  Primary  Secondary  Tertiary | Reference  0.55 (0.40 - 0.74)  0.21 (0.13 - 0.33) | Reference  0.86 (0.63 - 1.17)  0.43 (0.26 - 0.69) | Reference  0.85 (0.62 - 1.16)  0.42 (0.26 - 0.68) | Reference  0.88 (0.65 - 1.21)  0.46 (0.29 - 0.75) |
| **PSD-score x Education^b^**  Intermediate-PSD-scorexSecondary  Intermediate-PSD-scorexTertiary  High-PSD-scorexSecondary  High-PSD-scorexTertiary | 0.90 (0.55 - 1.45)  2.10 (1.11 - 3.98)  0.77 (0.44 - 1.33)  1.76 (0.87 - 3.56) | 0.98 (0.60 - 1.58)  2.15 (1.14 - 4.07)  0.86 (0.50 - 1.50)  1.80 (0.89 - 3.63) | 0.96 (0.59 - 1.56)  2.08 (1.10 - 3.95)  0.86 (0.50 - 1.50)  1.81 (0.89 - 3.65) | 0.96 (0.59 - 1.56)  2.08 (1.10 - 3.93)  0.85 (0.49 - 1.48)  1.69 (0.84 - 3.42) |
| **Age categories, years**  59 – 64  65 – 69  70 – 74  75 – 79  80 – 84  85 or above |  | Reference  2.82 (1.23 - 6.49)  5.21 (2.41 - 11.27)  8.63 (4.06 - 18.34)  25.18 (12.10 - 52.38)  62.19 (30.23 - 127.92) | Reference  2.82 (1.22 - 6.48)  5.19 (2.40 - 11.24)  8.60 (4.05 - 18.28)  25.05 (12.04 - 52.10)  61.53 (29.91 - 126.56) | Reference  2.54 (1.10 - 5.84)  4.09 (1.89 - 8.87)  5.27 (2.47 - 11.25)  11.22 (5.34 - 23.54)  17.33 (8.28 - 36.26) |
| **Sex**  Males  Females |  | Reference  0.40 (0.33 - 0.50) | Reference  0.40 (0.33 - 0.50) | Reference  0.43 (0.35 - 0.53) |
| **Country of birth**  Nordic  Non-Nordic |  | Reference  1.18 (0.86 - 1.61) | Reference  1.14 (0.84 - 1.57) | Reference  1.24 (0.90 - 1.70) |
| **Marital status**  Single  Married/registered partnership |  | Reference  0.92 (0.74 - 1.14) | Reference  0.92 (0.74 - 1.14) | Reference  1.27 (1.02 - 1.58) |
| **Income**  Low-income  Middle-income  High-income |  | Reference  0.87 (0.69 - 1.10)  0.79 (0.55 - 1.13) | Reference  0.86 (0.68 - 1.09)  0.78 (0.54 - 1.11) | Reference  0.95 (0.75 - 1.20)  0.91 (0.63 - 1.30) |
| **Housing tenure**  Rented  Tenant-owned  Owner-occupied |  | Reference  0.97 (0.77 - 1.22)  0.69 (0.53 - 0.89) | Reference  0.97 (0.77 - 1.23)  0.76 (0.58 - 1.00) | Reference  1.11 (0.88 - 1.40)  1.01 (0.77 - 1.33) |
| **Population density**  Low  Medium  High |  |  | Reference  1.14 (0.84 - 1.56)  1.50 (1.03 - 2.18) | Reference  1.16 (0.85 - 1.58)  1.59 (1.10 - 2.30) |
| **Receiving homecare**  No  Yes |  |  |  | Reference  6.83 (5.28 - 8.84) |
| **Cardiovascular diseases**  No  Yes |  |  |  | Reference  1.86 (1.39 - 2.49) |
| **Lung diseases**  No  Yes |  |  |  | Reference  1.78 (1.27 - 2.48) |
| **Endocrine diseases**  No  Yes |  |  |  | Reference  2.52 (1.85 - 3.43) |
| **Renal diseases**  No  Yes |  |  |  | Reference  1.50 (1.06 - 2.13) |
| **Stroke**  No  Yes |  |  |  | Reference  1.99 (1.28 - 3.09) |
| **Obesity**  No  Yes |  |  |  | Reference  1.52 (0.70 - 3.32) |
| **Cancer**  No  Yes |  |  |  | Reference  1.60 (1.21 - 2.30) |

^a^95% CI: 95% Confidence Interval.

^b^PSD-score x Education: moderation between PSD-score and education.

**Table A.8: The Cox regression models for effect moderation by income in the association between Perceived Sensory Dimension Score (PSD-score) and COVID-19 death among study participants during the study period 1 January to 31 December 2020 in Scania.**

| **Exposure variables** | **Model I (95% CI)^a^**  **N = 299156** | **Model II (95% CI)**  **N = 297990** | **Model III (95% CI)**  **N = 297985** | **Model IV (95% CI)**  **N = 297985** |
| --- | --- | --- | --- | --- |
| **PSD-score**  Low-PSD-score  Intermediate-PSD-score  High-PSD-score | Reference  0.87 (0.65 - 1.16)  0.63 (0.45 - 0.89) | Reference  0.77 (0.58 - 1.03)  0.65 (0.45 - 0.93) | Reference  0.88 (0.64 - 1.19)  0.83 (0.55 - 1.27) | Reference  0.93 (0.68 - 1.26)  0.89 (0.58 - 1.35) |
| **Income**  Low-income  Middle-income  High-income | Reference  0.65 (0.48 - 0.90)  0.21 (0.12 - 0.35) | Reference  0.89 (0.64 - 1.25)  0.70 (0.41 - 1.21) | Reference  0.89 (0.64 - 1.25)  0.70 (0.41 - 1.20) | Reference  1.00 (0.71 - 1.39)  0.87 (0.51 - 1.50) |
| **PSD-score x Income^b^**  Intermediate-PSD-scorexMiddle-income  Intermediate-PSD-scorexHigh-income  High-PSD-scorexMiddle-income  High-PSD-scorexHigh-income | 0.80 (0.49 - 1.29)  1.08 (0.53 - 2.23)  0.88 (0.51 - 1.50)  0.97 (0.44 - 2.12) | 0.90 (0.55 - 1.46)  1.28 (0.62 – 2.65)  1.06 (0.62 - 1.82)  1.12 (0.51 - 2.45) | 0.88 (0.54 - 1.44)  1.25 (0.60 – 2.57)  1.04 (0.60 - 1.78)  1.09 (0.50 - 2.40) | 0.86 (0.53 - 1.40)  1.19 (0.58 - 2.45)  1.00 (0.58 - 1.72)  0.95 (0.43 - 2.09) |
| **Age categories, years**  59 – 64  65 – 69  70 – 74  75 – 79  80 – 84  85 or above |  | Reference  2.82 (1.23 - 6.50)  5.21 (2.41 - 11.29)  8.67 (4.08 - 18.42)  25.31 (12.17 - 52.65)  62.48 (30.38 - 128.52) | Reference  2.82 (1.23 - 6.49)  5.20 (2.40 - 11.26)  8.64 (4.07 - 18.35)  25.17 (12.10 - 52.36)  61.80 (30.05 - 127.13) | Reference  2.53 (1.10 - 5.82)  4.10 (1.89 - 8.88)  5.29 (2.48 - 11.29)  11.27 (5.37 - 23.64)  17.39 (8.31 - 36.39) |
| **Sex**  Males  Females |  | Reference  0.40 (0.33 - 0.50) | Reference  0.40 (0.33 - 0.50) | Reference  0.43 (0.35 - 0.53) |
| **Country of birth**  Nordic  Non-Nordic |  | Reference  1.18 (0.86 - 1.62) | Reference  1.15 (0.84 - 1.57) | Reference  1.25 (0.91 - 1.71) |
| **Marital status**  Single  Married/registered partnership |  | Reference  0.92 (0.74 - 1.14) | Reference  0.92 (0.74 - 1.14) | Reference  1.27 (1.01 - 1.58) |
| **Education**  Primary  Secondary  Tertiary |  | Reference  0.83 (0.67 - 1.02)  0.66 (0.49 - 0.88) | Reference  0.82 (0.66 - 1.01)  0.64 (0.48 - 0.85) | Reference  0.85 (0.68 - 1.05)  0.69 (0.51 - 0.92) |
| **Housing tenure**  Rented  Tenant-owned  Owner-occupied |  | Reference  0.97 (0.77 - 1.23)  0.69 (0.53 - 0.89) | Reference  0.98 (0.77 - 1.23)  0.76 (0.58 - 1.00) | Reference  1.11 (0.88 - 1.41)  1.01 (0.77 - 1.33) |
| **Population density**  Low  Medium  High |  |  | Reference  1.14 (0.84 - 1.56)  1.51 (1.04 - 2.19) | Reference  1.16 (0.85 - 1.58)  1.61 (1.11 - 2.33) |
| **Receiving homecare**  No  Yes |  |  |  | Reference  6.83 (5.28 - 8.84) |
| **Cardiovascular diseases**  No  Yes |  |  |  | Reference  1.87 (1.39 - 2.49) |
| **Lung diseases**  No  Yes |  |  |  | Reference  1.78 (1.27 - 2.48) |
| **Endocrine diseases**  No  Yes |  |  |  | Reference  2.53 (1.86 - 3.43) |
| **Renal diseases**  No  Yes |  |  |  | Reference  1.51 (1.07 - 2.13) |
| **Stroke**  No  Yes |  |  |  | Reference  2.00 (1.29 - 3.11) |
| **Obesity**  No  Yes |  |  |  | Reference  1.50 (0.69 - 3.28) |
| **Cancer**  No  Yes |  |  |  | Reference  1.58 (1.09 - 2.28) |

^a^95% CI: 95% Confidence Interval.

^b^PSD-score x Income: moderation between PSD-score and income.

**Table A.9: The Cox regression models for effect moderation by education in the association between Perceived Sensory Dimension Score (PSD-score) and COVID-19 hospitalisation among study participants** **during the study period 1 January to 31 December 2020 in Scania.**

| **Exposure variables** | **Model I (95% CI)^a^**  **N = 298007** | **Model II (95% CI)**  **N = 297990** | **Model III (95% CI)**  **N = 297985** | **Model IV (95% CI)**  **N = 297985** |
| --- | --- | --- | --- | --- |
| **PSD-score**  Low-PSD-score  Intermediate-PSD-score  High-PSD-score | Reference  0.64 (0.53 - 0.79)  0.47 (0.38 - 0.59) | Reference  0.74 (0.61 - 0.91)  0.68 (0.54 - 0.86) | Reference  0.87 (0.71 - 1.08)  0.89 (0.68 - 1.17) | Reference  0.92 (0.74 - 1.14)  0.95 (0.73 - 1.24) |
| **Education**  Primary  Secondary  Tertiary | Reference  0.77 (0.65 - 0.91)  0.49 (0.40 - 0.61) | Reference  0.93 (0.78 - 1.10)  0.70 (0.56 - 0.86) | Reference  0.92 (0.78 - 1.10)  0.68 (0.55 - 0.85) | Reference  0.99 (0.83 - 1.18)  0.75 (0.61 - 0.93) |
| **PSD-scorexEducation^b^**  Intermediate-PSD-scorexSecondary  Intermediate-PSD-scorexTertiary  High-PSD-scorexSecondary  High-PSD-scorexTertiary | 0.91 (0.69 - 1.20)  1.03 (0.74 - 1.43)  0.89 (0.65 - 1.21)  1.10 (0.76 - 1.57) | 0.97 (0.74 - 1.27)  1.03 (0.74 - 1.44)  0.96 (0.70 - 1.30)  1.10 (0.76 - 1.57) | 0.95 (0.72 - 1.25)  0.99 (0.71 - 1.38)  0.96 (0.70 - 1.30)  1.10 (0.77 - 1.58) | 0.92 (0.70 - 1.21)  0.95 (0.68 - 1.33)  0.91 (0.67 - 1.24)  1.04 (0.73 - 1.49) |
| **Age categories, years**  59 – 64  65 – 69  70 – 74  75 – 79  80 – 84  85 or above |  | Reference  1.22 (1.01 - 1.49)  1.20 (0.98 - 1.47)  1.81 (1.49 - 2.20)  2.87 (2.36 - 3.50)  4.58 (3.79 - 5.55) | Reference  1.22 (1.01 - 1.48)  1.20 (0.98 - 1.46)  1.81 (1.49 - 2.20)  2.87 (2.35 - 3.49)  4.55 (3.76 - 5.51) | Reference  1.16 (0.95 - 1.40)  1.05 (0.86 - 1.29)  1.39 (1.14 - 1.69)  1.82 (1.48 - 2.23)  2.14 (1.74 - 2.64) |
| **Sex**  Males  Females |  | Reference  0.54 (0.48 - 0.60) | Reference  0.54 (0.48 - 0.60) | Reference  0.59 (0.52 - 0.66) |
| **Country of birth**  Nordic  Non-Nordic |  | Reference  2.47 (2.17 - 2.81) | Reference  2.38 (2.09 - 2.71) | Reference  2.48 (2.18 - 2.83) |
| **Marital status**  Single  Married/registered partnership |  | Reference  1.02 (0.91 - 1.15) | Reference  1.03 (0.91 - 1.16) | Reference  1.16 (1.03 - 1.31) |
| **Income**  Low income  Middle income  High income |  | Reference  0.90 (0.79 - 1.03)  0.85 (0.71 - 1.00) | Reference  0.90 (0.79 - 1.03)  0.83 (0.70 - 0.99) | Reference  0.99 (0.87 - 1.13)  0.95 (0.80 - 1.13) |
| **Housing tenure**  Rented  Tenant-owned  Owner-occupied |  | Reference  0.75 (0.66 - 0.86)  0.60 (0.52 - 0.70) | Reference  0.76 (0.67 - 0.87)  0.69 (0.59 - 0.80) | Reference  0.83 (0.72 - 0.95)  0.80 (0.69 - 0.93) |
| **Living with children**  No  Yes |  | Reference  2.70 (2.02 - 3.61) | Reference  2.68 (2.01 - 3.59) | Reference  2.94 (2.19 - 3.94) |
| **Population density**  Low  Medium  High |  |  | Reference  1.05 (0.88 - 1.25)  1.57 (1.28 - 1.93) | Reference  1.07 (0.89 - 1.27)  1.63 (1.33 - 2.01) |
| **Receiving homecare**  No  Yes |  |  |  | Reference  2.80 (2.42 - 3.23) |
| **Cardiovascular diseases**  No  Yes |  |  |  | Reference  2.57 (2.13 - 3.10) |
| **Lung diseases**  No  Yes |  |  |  | Reference  2.08 (1.69 - 2.57) |
| **Endocrine diseases**  No  Yes |  |  |  | Reference  2.14 (1.75 - 2.63) |
| **Renal diseases**  No  Yes |  |  |  | Reference  1.49 (1.19 - 1.87) |
| **Stroke**  No  Yes |  |  |  | Reference  1.17 (0.82 - 1.67) |
| **Obesity**  No  Yes |  |  |  | Reference  1.77 (1.17 - 2.68) |
| **Cancer**  No  Yes |  |  |  | Reference  1.66 (1.31 - 2.10) |

^a^95% CI: 95% Confidence Interval.

^b^PSD-score x Education: moderation between PSD-score and education.

**Table A.10: The Cox regression models for effect moderation by income in the association between Perceived Sensory Dimension Score (PSD-score) and COVID-19 hospitalisation among study participants during the study period 1 January to 31 December 2020 in Scania.**

| **Exposure variables** | **Model I (95% CI)^a^**  **N = 299156** | **Model II (95% CI)**  **N = 297990** | **Model III (95% CI)**  **N = 297985** | **Model IV (95% CI)**  **N = 297985** |
| --- | --- | --- | --- | --- |
| **PSD-score**  Low-PSD-score  Intermediate-PSD-score  High-PSD-score | Reference  0.62 (0.52 - 0.74)  0.46 (0.37 - 0.56) | Reference  0.70 (0.59 - 0.84)  0.65 (0.52 – 0.80 | Reference  0.82 (0.69 - 0.99)  0.86 (0.67 - 1.11) | Reference  0.87 (0.72 - 1.04)  0.92 (0.71 - 1.18) |
| **Income**  Low income  Middle income  High income | Reference  0.61 (0.51 - 0.73)  0.40 (0.33 - 0.50) | Reference  0.82 (0.69 - 1.00)  0.86 (0.68 - 1.08) | Reference  0.83 (0.69 - 1.00)  0.86 (0.68 - 1.08) | Reference  0.94 (0.78 - 1.13)  1.02 (0.80 - 1.28) |
| **PSD-score x Income^b^**  Intermediate-PSD-scorexMiddle income  Intermediate-PSD-scorexHigh income  High PSD-scorexMiddle income  High PSD-scorexHigh Income | 1.22 (0.93 - 1.61)  0.96 (0.69 - 1.35)  1.17 (0.85 - 1.60)  1.21 (0.86 - 1.72) | 1.21 (0.92 - 1.60)  0.92 (0.65 - 1.29)  1.14 (0.83 - 1.56)  1.07 (0.75 - 1.52) | 1.19 (0.91 - 1.57)  0.89 (0.63 - 1.25)  1.10 (0.80 - 1.51)  1.03 (0.73 - 1.47) | 1.15 (0.87 - 1.52)  0.84 (0.60 - 1.18)  1.05 (0.77 - 1.45)  0.95 (0.67 - 1.35) |
| **Age categories, years**  59 – 64  65 – 69  70 – 74  75 – 79  80 – 84  85 or above |  | Reference  1.22 (1.01 - 1.49)  1.20 (0.98 - 1.46)  1.81 (1.49 - 2.20)  2.87 (2.36 - 3.50)  4.59 (3.79 - 5.55) | Reference  1.22 (1.01 - 1.48)  1.19 (0.98 - 1.46)  1.81 (1.48 - 2.20)  2.87 (2.35 - 3.49)  4.56 (3.76 - 5.51) | Reference  1.16 (0.95 - 1.41)  1.05 (0.86 - 1.29)  1.39 (1.14 - 1.69)  1.82 (1.48 - 2.23)  2.14 (1.74 - 2.64) |
| **Sex**  Males  Females |  | Reference  0.54 (0.48 - 0.60) | Reference  0.54 (0.48 - 0.60) | Reference  0.59 (0.52 - 0.66) |
| **Country of birth**  Nordic  Non-Nordic |  | Reference  2.47 (2.17 - 2.81) | Reference  2.38 (2.09 - 2.71) | Reference  2.48 (2.18 - 2.83) |
| **Marital status**  Single  Married/registered partnership |  | Reference  1.02 (0.91 - 1.15) | Reference  1.03 (0.91 - 1.16) | Reference  1.16 (1.03 - 1.31) |
| **Education**  Primary  Secondary  Tertiary |  | Reference  0.91 (0.81 - 1.03)  0.72 (0.62 - 0.84) | Reference  0.90 (0.80 - 1.02)  0.70 (0.60 - 0.81) | Reference  0.95 (0.84 - 1.07)  0.75 (0.64 - 0.87) |
| **Housing tenure**  Rented  Tenant-owned  Owner-occupied |  | Reference  0.75 (0.66 - 0.86)  0.60 (0.52 - 0.70) | Reference  0.76 (0.67 - 0.87)  0.69 (0.59 - 0.80) | Reference  0.83 (0.73 - 0.95)  0.80 (0.69 - 0.93) |
| **Living with children**  No  Yes |  | Reference  2.70 (2.01 - 3.61) | Reference  2.69 (2.01 - 3.59) | Reference  2.95 (2.20 - 3.95) |
| **Population density**  Low  Medium  High |  |  | Reference  1.05 (0.88 - 1.25)  1.57 (1.27 - 1.93) | Reference  1.06 (0.89 - 1.27)  1.63 (1.33 - 2.00) |
| **Receiving homecare**  No  Yes |  |  |  | Reference  2.80 (2.42 - 3.23) |
| **Cardiovascular diseases**  No  Yes |  |  |  | Reference  2.57 (2.12 - 3.10) |
| **Lung diseases**  No  Yes |  |  |  | Reference  2.09 (1.69 - 2.57) |
| **Endocrine diseases**  No  Yes |  |  |  | Reference  2.14 (1.75 - 2.63) |
| **Renal diseases**  No  Yes |  |  |  | Reference  1.49 (1.19 - 1.87) |
| **Stroke**  No  Yes |  |  |  | Reference  1.16 (0.82 - 1.66) |
| **Obesity**  No  Yes |  |  |  | Reference  1.77 (1.17 - 2.69) |
| **Cancer**  No  Yes |  |  |  | Reference  1.65 (1.30 - 2.09) |

^a^95% CI: 95% Confidence Interval.

^b^PSD-scorex Income: moderation between PSD-score and income.

**Table A.11: Cox regression models for the associations between Perceived Sensory Dimension Score (PSD-score) and COVID-19 death among study participants during the study period 1 January to 19 November 2020 in Scania.**

| **Exposure variables** | **Model I (95% CI)^a^**  **N = 299156** | **Model II (95% CI)**  **N = 297990** | **Model III (95% CI)**  **N = 297985** | **Model IV (95% CI)**  **N = 297985** |
| --- | --- | --- | --- | --- |
| **PSD-score**  Low-PSD-score  Intermediate-PSD-score  High-PSD-score | Reference  0.83 (0.61 - 1.13)  0.61 (0.43 - 0.86) | Reference  0.84 (0.61 - 1.15)  0.83 (0.57 - 1.21) | Reference  0.95 (0.67 - 1.35)  1.06 (0.66 - 1.73) | Reference  0.99 (0.70 - 1.41))  1.10 (0.68 - 1.80) |
| **Age categories, years**  59 – 64  65 – 69  70 – 74  75 – 79  80 – 84  85 or above |  | Reference  1.97 (0.64 - 6.03)  3.84 (1.41 - 10.48)  5.30 (1.97 - 14.23)  19.15 (7.51 - 48.87)  45.57 (18.16 - 114.31) | Reference  1.97 (0.64 - 6.01)  3.83 (1.41 - 10.46)  5.29 (1.97 - 14.20)  19.10 (7.49 - 48.74)  45.23 (18.03 - 113.46) | Reference  1.76 (0.57 - 5.37)  2.99 (1.09 - 8.17)  3.23 (1.19 - 8.73)  8.52 (3.29 - 22.04)  12.78 (4.96 - 32.97) |
| **Sex**  Males  Females |  | Reference  0.38 (0.28 - 0.51) | Reference  0.38 (0.28 - 0.50) | Reference  0.41 (0.30 - 0.55) |
| **Country of birth**  Nordic  Non-Nordic |  | Reference  1.13 (0.72 - 1.79) | Reference  1.10 (0.69 - 1.73) | Reference  1.19 (0.75 - 1.88) |
| **Marital status**  Single  Married/registered partnership |  | Reference  0.85 (0.62 - 1.15) | Reference  0.85 (0.63 - 1.16) | Reference  1.16 (0.85 - 1.59) |
| **Education**  Primary  Secondary  Tertiary |  | Reference  0.77 (0.57 - 1.05)  0.80 (0.54 - 1.18) | Reference  0.76 (0.56 - 1.04)  0.77 (0.52 - 1.15) | Reference  0.78 (0.57 - 1.07)  0.84 (0.57 - 1.25) |
| **Income**  Low income  Middle income  High income |  | Reference  0.89 (0.64 - 1.24)  0.63 (0.37 - 1.07) | Reference  0.89 (0.64 - 1.23)  0.62 (0.36 - 1.05) | Reference  0.96 (0.69 - 1.34)  0.73 (0.43 - 1.24) |
| **Housing tenure**  Rented  Tenant-owned  Owner-occupied |  | Reference  1.06 (0.76 - 1.48)  0.67 (0.46 - 0.97) | Reference  1.07 (0.77 - 1.49)  0.74 (0.50 - 1.09) | Reference  1.20 (0.86 - 1.68)  0.97 (0.66 - 1.43) |
| **Population density**  Low  Medium  High |  |  | Reference  1.07 (0.69 - 1.65)  1.48 (0.88 - 2.50) | Reference  1.10 (0.72 - 1.70)  1.60 (0.95 - 2.69) |
| **Receiving homecare**  No  Yes |  |  |  | Reference  6.45 (4.47 - 9.30) |
| **Cardiovascular diseases**  No  Yes |  |  |  | Reference  2.17 (1.46 - 3.23) |
| **Lung diseases**  No  Yes |  |  |  | Reference  1.29 (0.79 - 2.10) |
| **Endocrine diseases**  No  Yes |  |  |  | Reference  2.41 (1.57 - 3.70) |
| **Renal diseases**  No  Yes |  |  |  | Reference  1.24 (0.75 - 2.03) |
| **Stroke**  No  Yes |  |  |  | Reference  2.00 (1.09 - 3.69) |
| **Obesity**  No  Yes |  |  |  | Reference  3.04 (1.28 - 7.20) |
| **Cancer**  No  Yes |  |  |  | Reference  1.94 (1.21 - 3.13) |

^a^95% CI: 95% Confidence Interval.

**Table A.12: Cox regression models for the associations between Perceived Sensory Dimension Score (PSD-score) and COVID-19 death among study participants during the study period from 20 November to 31 December 2020.**

| **Exposure variables** | **Model I (95% CI)^a^**  **N =** 292963 | **Model II (95% CI)**  **N =** 291823 | **Model III (95% CI)**  **N =** 291818 | **Model IV (95% CI)**  **N =** 291818 |
| --- | --- | --- | --- | --- |
| **PSD-score**  Low-PSD-score  Intermediate-PSD-score  High-PSD-score | Reference  0.71 (0.53 - 0.97)  0.45 (0.31 - 0.64) | Reference  0.70 (0.51 - 0.96)  0.54 (0.36 - 0.79) | Reference  0.79 (0.56 - 1.10)  0.69 (0.42 - 1.13) | Reference  0.82 (0.58 - 1.15)  0.70 (0.43 - 1.15) |
| **Age categories, years**  59 – 64  65 – 69  70 – 74  75 – 79  80 – 84  85 or above |  | Reference  4.28 (1.18 - 15.55)  7.58 (2.22 - 25.86)  14.20 (4.28 - 47.12)  35.72 (10.96 - 116.43)  90.67 (28.21 - 291.45) | Reference  4.27 (1.17 - 15.52)  7.56 (2.21 - 25.78)  14.13 (4.26 - 46.87)  35.43 (10.87 - 115.50)  89.42 (27.82 - 287.45) | Reference  3.86 (1.06 - 14.02)  5.98 (1.75 - 20.45)  8.76 (2.63 - 29.20)  16.15 (4.90 - 53.19)  25.56 (7.79 - 83.88) |
| **Sex**  Males  Females |  | Reference  0.43 (0.32 - 0.58) | Reference  0.43 (0.32 - 0.58) | Reference  0.44 (0.33 - 0.60) |
| **Country of birth**  Nordic  Non-Nordic |  | Reference  1.25 (0.81 - 1.92) | Reference  1.21 (0.78 - 1.87) | Reference  1.32 (0.86 - 2.05) |
| **Marital status**  Single  Married/registered partnership |  | Reference  0.98 (0.72 - 1.33) | Reference  0.98 (0.72 - 1.34) | Reference  1.37 (1.00 - 1.88) |
| **Education**  Primary  Secondary  Tertiary |  | Reference  0.88 (0.65 - 1.18)  0.53 (0.34 - 0.82) | Reference  0.86 (0.64 - 1.17))  0.77 (0.52 - 1.15) | Reference  0.90 (0.67 - 1.21)  0.56 (0.36 - 0.86) |
| **Income**  Low income  Middle income  High income |  | Reference  0.84 (0.60 - 1.18)  0.97 (0.60 - 1.57) | Reference  0.83 (0.60 - 1.17)  0.95 (0.58 - 1.53) | Reference  0.91 (0.65 - 1.28)  1.11 (0.69 - 1.81) |
| **Housing tenure**  Rented  Tenant-owned  Owner-occupied |  | Reference  0.89 (0.64 - 1.25)  0.72 (0.50 - 1.03) | Reference  0.89 (0.64 - 1.25)  0.80 (0.54 - 1.17) | Reference  1.03 (0.74 - 1.44)  1.08 (0.73 - 1.58) |
| **Population density**  Low  Medium  High |  |  | Reference  1.22 (0.77 - 1.91)  1.56 (0.92 - 2.66) | Reference  1.22 (0.78 - 1.91)  1.64 (0.96 - 2.78) |
| **Receiving homecare**  No  Yes |  |  |  | Reference  7.09 ((4.94 - 10.17) |
| **Cardiovascular diseases**  No  Yes |  |  |  | Reference  1.53 (0.99 - 2.34) |
| **Lung diseases**  No  Yes |  |  |  | Reference  2.44 (1.53 - 3.87) |
| **Endocrine diseases**  No  Yes |  |  |  | Reference  2.78 (1.80 - 4.30) |
| **Renal diseases**  No  Yes |  |  |  | Reference  1.80 (1.10 - 2.93) |
| **Stroke**  No  Yes |  |  |  | Reference  2.04 (1.08 - 3.87) |
| **Obesity**  No  Yes |  |  |  | Reference  0.35 (0.05 - 2.60) |
| **Cancer**  No  Yes |  |  |  | Reference  1.20 (0.68 - 2.14) |

^a^95% CI: 95% Confidence Interval.

**Table A.13: Cox regression models for the associations between Perceived Sensory Dimension Score (PSD-score) and COVID-19 hospitalisation among study participants during the study period from 01 January to 20 November 2020 in Scania.**

| **Exposure variables** | **Model I (95% CI)^a^**  **N = 299156** | **Model II (95% CI)**  **N = 297990** | **Model III (95% CI)**  **N = 297985** | **Model IV (95% CI)**  **N = 297985** |
| --- | --- | --- | --- | --- |
| **PSD-score**  Low-PSD-score  Intermediate-PSD-score  High-PSD-score | Reference  0.60 (0.51 - 0.72)  0.50 (0.41 - 0.60) | Reference  0.72 (0.60 - 0.86)  0.78 (0.63- 0.96) | Reference  0.82 (0.67 - 0.99)  1.00 (0.76 - 1.31) | Reference  0.85 (0.70 - 1.03)  1.04 (0.79 - 1.36) |
| **Age categories, years**  59 – 64  65 – 69  70 – 74  75 – 79  80 – 84  85 or above |  | Reference  1.18 (0.90 - 1.55)  1.21 (0.91 - 1.60)  1.53 (1.15 - 2.02)  2.76 (2.10 - 3.64)  4.00 (3.05 - 5.23) | Reference  1.18 (0.90 - 1.55)  1.21 (0.91 - 1.60)  1.52 (1.15 - 2.02)  2.76 (2.09 - 3.63)  3.97 (3.03 - 5.20) | Reference  1.10 (0.84 - 1.45)  1.02 (0.77 - 1.36)  1.10 (0.83 - 1.47)  1.59 (1.20 - 2.12)  1.65 (1.23 - 2.22) |
| **Sex**  Males  Females |  | Reference  0.59 (0.50 - 0.68) | Reference  0.59 (0.50 - 0.68) | Reference  0.65 (0.55 - 0.76) |
| **Country of birth**  Nordic  Non-Nordic |  | Reference  2.56 (2.13 - 3.08) | Reference  2.47 (2.06 - 2.97) | Reference  2.60 (2.16 - 3.12) |
| **Marital status**  Single  Married/registered partnership |  | Reference  1.07 (0.90 - 1.26) | Reference  1.07 (0.91 - 1.27) | Reference  1.24 (1.05 - 1.47) |
| **Education level**  Primary  Secondary  Tertiary |  | Reference  0.93 (0.78 - 1.10)  0.73 (0.59 - 0.91) | Reference  0.92 (0.77 - 1.09)  0.71 (0.57 - 0.89) | Reference  0.97 (0.82 - 1.15)  0.78 (0.63 - 0.97) |
| **Income level**  Low income  Middle income  High income |  | Reference  0.87 (0.72 - 1.04)  0.73 (0.57 - 0.94) | Reference  0.86 (0.72 - 1.04)  0.72 (0.56 - 0.93) | Reference  0.97 (0.81 - 1.17)  0.85 (0.66 - 1.09) |
| **Housing tenure**  Rented  Tenant-owned  Owner-occupied |  | Reference  0.72 (0.59 - 0.87)  0.59 (0.48 - 0.72) | Reference  0.73 (0.60 - 0.88)  0.66 (0.53 - 0.82) | Reference  0.80 (0.66 - 0.97)  0.79 (0.63 - 0.98) |
| **Living with children**  No  Yes |  | Reference  2.04 (1.30 - 3.20) | Reference  2.03 (1.29 - 3.19) | Reference  2.27 (1.44 - 3.57) |
| **Population density**  Low  Medium  High |  |  | Reference  1.05 (0.82 - 1.35)  1.52 (1.13 - 2.04) | Reference  1.08 (0.84 - 1.38)  1.58 (1.18 - 2.12) |
| **Receiving homecare**  No  Yes |  |  |  | Reference  3.25 (2.65 - 3.98) |
| **Cardiovascular diseases**  No  Yes |  |  |  | Reference  2.96 (2.30 - 3.83) |
| **Lung diseases**  No  Yes |  |  |  | Reference  2.24 (1.70 - 2.96) |
| **Endocrine diseases**  No  Yes |  |  |  | Reference  1.86 (1.41 - 2.45) |
| **Renal diseases**  No  Yes |  |  |  | Reference  1.25 (0.91 - 1.71) |
| **Stroke**  No  Yes |  |  |  | Reference  1.16 (0.73 - 1.86) |
| **Obesity**  No  Yes |  |  |  | Reference  2.80 (1.75 - 4.48) |
| **Cancer**  No  Yes |  |  |  | Reference  1.73 (1.27 - 2.37) |

^a^95% CI: 95% Confidence Interval.

**Table A.14: Cox regression models for the associations between Perceived Sensory Dimension Score (PSD-score) and COVID-19 hospitalisation among study participants during the study period from 21 November to 31 December 2020 in Scania.**

| **Exposure variables** | **Model I (95% CI)^a^**  **N =** 292426 | **Model II (95% CI)**  **N =** 291291 | **Model III (95% CI)**  **N =** 291286 | **Model IV (95% CI)**  **N =** 291286 |
| --- | --- | --- | --- | --- |
| **PSD-score**  Low-PSD-score  Intermediate-PSD-score  High-PSD-score | Reference  0.66 (0.56 - 0.78)  0.42 (0.34 - 0.51) | Reference  0.76 (0.64 - 0.91)  0.61 (0.49 - 0.76) | Reference  0.89 (0.74 - 1.08)  0.81 (0.62 - 1.07) | Reference  0.92 (0.76 - 1.11)  0.83 (0.63 - 1.10) |
| **Age categories, years**  59 – 64  65 – 69  70 – 74  75 – 79  80 – 84  85 or above |  | Reference  1.26 (0.95 - 1.66)  1.15 (0.85 - 1.54)  2.12 (1.62 - 2.79)  2.93 (2.20 - 3.89)  5.24 (4.00 - 6.87) | Reference  1.25 (0.95 - 1.65)  1.14 (0.85 - 1.54)  2.12 (1.61 - 2.78)  2.92 (2.19 - 3.88)  5.20 (3.97 - 6.81) | Reference  1.20 (0.91 - 1.58)  1.03 (0.77 - 1.39)  1.72 (1.30 - 2.27)  2.02 (1.51 - 2.71)  2.78 (2.07 - 3.74) |
| **Sex**  Males  Females |  | Reference  0.50 (0.42 - 0.58) | Reference  0.50 (0.42 - 0.58) | Reference  0.54 (0.46 - 0.63) |
| **Country of birth**  Nordic  Non-Nordic |  | Reference  2.43 (2.02 - 2.92) | Reference  2.33 (1.94 - 2.81) | Reference  2.42 (2.01 - 2.92) |
| **Marital status**  Single  Married/registered partnership |  | Reference  0.97 (0.82 - 1.15) | Reference  0.98 (0.83 - 1.16) | Reference  1.08 (0.91 - 1.28) |
| **Education level**  Primary  Secondary  Tertiary |  | Reference  0.89 (0.75 - 1.05)  0.70 (0.56 - 0.86) | Reference  0.87 (0.74 - 1.04)  0.67 (0.54 - 0.83) | Reference  0.91 (0.77 - 1.08)  0.72 (0.58 - 0.89) |
| **Income level**  Low income  Middle income  High income |  | Reference  0.96 (0.79 - 1.15)  0.99 (0.78 - 1.26) | Reference  0.95 (0.79 - 1.15)  0.97 (0.76 - 1.23) | Reference  1.03 (0.85 - 1.24)  1.08 (0.85 - 1.38) |
| **Housing tenure**  Rented  Tenant-owned  Owner-occupied |  | Reference  0.78 (0.65 - 0.95)  0.62 (0.51 - 0.76) | Reference  0.79 (0.66 - 0.96)  0.72 (0.58 - 0.90) | Reference  0.86 (0.71 - 1.04)  0.82 (0.66 - 1.02) |
| **Living with children**  No  Yes |  | Reference  3.46 (2.36 - 5.07) | Reference  3.44 (2.35 - 5.05) | Reference  3.70 (2.52 - 5.44) |
| **Population density**  Low  Medium  High |  |  | Reference  1.06 (0.83 - 1.37)  1.64 (1.22 - 2.20) | Reference  1.07 (0.83 - 1.38)  1.70 (1.27 - 2.28) |
| **Receiving homecare**  No  Yes |  |  |  | Reference  2.36 (1.92 - 2.90) |
| **Cardiovascular diseases**  No  Yes |  |  |  | Reference  2.21 (1.67 - 2.93) |
| **Lung diseases**  No  Yes |  |  |  | Reference  1.81 (1.31 - 2.50) |
| **Endocrine diseases**  No  Yes |  |  |  | Reference  2.50 (1.85 - 3.38) |
| **Renal diseases**  No  Yes |  |  |  | Reference  1.87 (1.34 - 2.61) |
| **Stroke**  No  Yes |  |  |  | Reference  1.18 (0.68 - 2.04) |
| **Obesity**  No  Yes |  |  |  | Reference  0.61 (0.22 - 1.67) |
| **Cancer**  No  Yes |  |  |  | Reference  1.49 (1.04 - 2.14) |

^a^95% CI: 95% Confidence Interval.

**Table A.15: Cox regression analysis for the association between Perceived Sensory Dimension Score (PSD-score) and COVID-19 death stratified according to population density.**

|  | **Low-population density**  **HR^a^ (95% CI)^b^**  **N = 95040** | **Medium-population density**  **HR (95% CI)**  **N = 101606** | **High-population density**  **HR (95% CI)**  **N = 101339** |
| --- | --- | --- | --- |
| **PSD-score exposure** |  |  |  |
| Low PSD-score | Reference | Reference | Reference |
| Intermediate PSD-score | 0.87 (0.32 – 2.36) | 0.67 (0.45 - 1.00) | 1.08 (0.79 - 1.48) |
| High PSD-score | 0.92 (0.35 – 2.39) | 0.65 (0.40 - 1.05) | 3.07 (1.10 - 8.56) |

^a^Hazard Ratios

^b^95% CI: 95% Confidence Interval.

The Hazard Ratios are adjusted for age, sex, marital status, place of birth, income, housing tenure, population density, receiving homecare services and comorbidities (cancer, renal diseases, lung diseases, stroke, endocrine diseases, cardiovascular diseases, and obesity).

**Table A.16: Cox regression analysis for the association between Perceived Sensory Dimension Score (PSD-score) and COVID-19 hospitalisation stratified according to population density.**

|  | **Low-population density**  **HR^a^ (95% CI)^b^**  **N = 95040** | **Medium-population density**  **HR (95% CI)**  **N = 101606** | **High-population density**  **HR (95% CI)**  **N = 101339** |
| --- | --- | --- | --- |
| **PSD-score** |  |  |  |
| Low PSD-score | Reference | Reference | Reference |
| Intermediate PSD-score | 0.70 (0.41– 1.18) | 0.88 (0.69- 1.12) | 0.92 (0.77 - 1.10) |
| High PSD-score | 0.79 (0.48 – 1.30) | 0.84 (0.63 - 1.12) | 1.43 (0.67 – 3.05) |

^a^Hazard Ratios

^b^95% CI: 95% Confidence Interval.

The Hazard Ratios are adjusted for age, sex, marital status, place of birth, income, housing tenure, living with children, population density, receiving homecare services and comorbidities (cancer, renal diseases, lung diseases, stroke, endocrine diseases, cardiovascular diseases, and obesity).
